# Design of chimeric GLP-1A using oligomeric bile acids to utilize transporter-mediated endocytosis for oral delivery

**DOI:** 10.1186/s40824-023-00421-7

**Published:** 2023-09-02

**Authors:** Seho Kweon, Jun-Hyuck Lee, Seong-Bin Yang, Seong Jin Park, Laxman Subedi, Jung-Hyun Shim, Seung-Sik Cho, Jeong Uk Choi, Youngro Byun, Jooho Park, Jin Woo Park

**Affiliations:** 1 Department of Molecular Medicine and Biopharmaceutical Science, Graduate School of Convergence Science and Technology, Seoul National University, Seoul, 08826 Republic of Korea; 2 Biomedicine Cutting Edge Formulation Technology Center, Mokpo National University, Jeonnam, 58554 Republic of Korea; 3 Department of Applied Life Science, Graduate School, BK21 Program, Konkuk University, Chungju, 27478 Republic of Korea; 4 College of Pharmacy, Seoul National University, Seoul, 08826 Republic of Korea; 5 Department of Biomedicine, Health & Life Convergence Sciences, BK21 Four, Biomedical and Healthcare Research Institute, Mokpo National University, Jeonnam, 58554 Republic of Korea; 6 College of Pharmacy and Natural Medicine Research Institute, Mokpo National University, Jeonnam, 58554 Republic of Korea; 7 College of Pharmacy, Research Institute of Pharmaceutical Sciences, Chonnam National University, Gwangju, 61186 Republic of Korea

**Keywords:** Chimeric peptide, Oral GLP-1 agonist, Oligomeric bile acids, In silico molecular docking, ASBT-mediated endocytosis

## Abstract

**Background:**

Despite the effectiveness of glucagon-like peptide-1 agonist (GLP-1A) in the treatment of diabetes, its large molecular weight and high hydrophilicity result in poor cellular permeability, thus limiting its oral bioavailability. To address this, we developed a chimeric GLP-1A that targets transporter-mediated endocytosis to enhance cellular permeability to GLP-1A by utilizing the transporters available in the intestine, particularly the apical sodium-dependent bile acid transporter (ASBT).

**Methods:**

In silico molecular docking and molecular dynamics simulations were used to investigate the binding interactions of *mono*-, *bis*-, and *tetra*-deoxycholic acid (DOCA) (*mono*DOCA, *bis*DOCA, and *tetra*DOCA) with ASBT. After synthesizing the chimeric GLP-1A-conjugated oligomeric DOCAs (*m*D-G1A, *b*D-G1A, and *t*D-G1A) using a maleimide reaction, in vitro cellular permeability and insulinotropic effects were assessed. Furthermore, in vivo oral absorption in rats and hypoglycemic effect on diabetic *db/db* mice model were evaluated.

**Results:**

In silico results showed that *tetra*DOCA had the lowest interaction energy, indicating high binding affinity to ASBT. Insulinotropic effects of GLP-1A-conjugated oligomeric DOCAs were not different from those of GLP-1A-Cys or exenatide. Moreover, *b*D-G1A and *t*D-G1A exhibited improved in vitro Caco-2 cellular permeability and showed higher in vivo bioavailability (7.58% and 8.63%) after oral administration. Regarding hypoglycemic effects on *db/db* mice, *t*D-G1A (50 μg/kg) lowered the glucose level more than *b*D-G1A (50 μg/kg) compared with the control (35.5% vs. 26.4%).

**Conclusion:**

GLP-1A was conjugated with oligomeric DOCAs, and the resulting chimeric compound showed the potential not only for glucagon-like peptide-1 receptor agonist activity but also for oral delivery. These findings suggest that oligomeric DOCAs can be used as effective carriers for oral delivery of GLP-1A, offering a promising solution for enhancing its oral bioavailability and improving diabetes treatment.

**Graphical Abstract:**

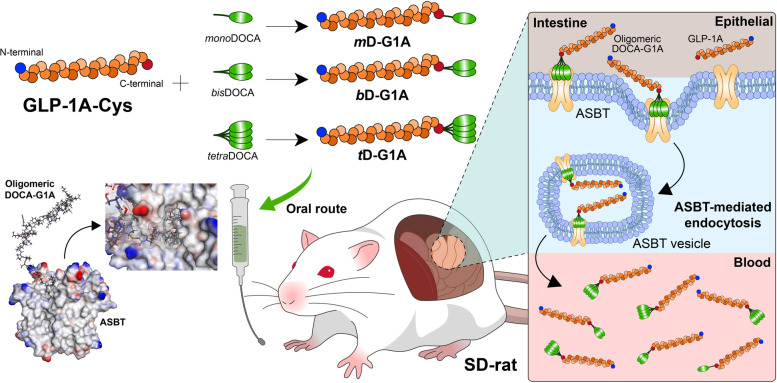

**Supplementary Information:**

The online version contains supplementary material available at 10.1186/s40824-023-00421-7.

## Background

Diabetes is a representative chronic disease that requires lifetime management. Because of the need for frequent and combination dosing, most hypoglycemic therapeutics are small molecule-based oral drugs. Therapeutic peptides such as insulin are highly effective in patients with worsening diabetes, but the patient’s condition and medications must be cautiously monitored because of the risk of hypoglycemia [1, 2]. By contrast, glucagon-like peptide-1 agonist (GLP-1A) has shown a strong glucose-dependent hypoglycemic effect with few cardiovascular-related side effects, resulting in a significant market share [3–5]. However, the applications of GLP-1A are hampered by its short half-life, and its administration routes are limited by its low stability [6, 7]. Various efforts have been made to overcome these weaknesses. The half-life of GLP-1A was successfully extended to match that of exenatide (2.4 h) and liraglutide (13 h), and subsequently that of dulaglutide (5 days) and semaglutide (7 days) [8]. Various dual/triple agonists for glucagon-like peptide-1 receptor (GLP-1R), which also target the gastric inhibitory peptide receptor and glucagon receptor, have recently been explored to increase treatment efficacy for both diabetes and obesity in the clinical setting [9, 10]. However, the development of oral GLP-1A is challenging; semaglutide (Rybelsus) is the only new-era oral GLP-1A on the market [11–13]. Frequent injection can be burdensome for patients even if treated once weekly; most patients prefer oral to injectable drugs [14]. Moreover, for patients taking other oral combination drugs, the additional use of injectable drugs results in low therapeutic adherence and compliance [15].

The poor cellular permeability and stability of peptides in the gastrointestinal (GI) tract represent the main hurdles to the development of oral GLP-1A [16, 17]. Peptide engineering resulting in substitutions, alpha-methylation, and bis-lipidation is used to protect the vulnerable amino acid sequence of GLP-1A against degradation by dipeptidyl peptidase-4 and GI enzymes [18–20]. This approach also improves peptide stability in the GI tract and allows raw GLP-1A to contact the intestinal epithelial surface, resulting in increased oral absorption [21]. However, because of the hydrophilic and macromolecular properties of GLP-1A, penetration of the intestinal mucous and cellular layers remains difficult. To address this issue, drug delivery systems for oral peptides have been developed to improve the cellular permeability of peptides using absorption enhancers [e.g., sodium N-[8-(2-hydroxybenzoyl)amino]caprylate (SNAC), bile acids, β-cyclodextrin, and cell-penetrating peptides] and ingestible devices [e.g., self-orienting millimeter-scale applicator (SOMA), liquid-injecting SOMA, luminal unfolding microneedle injector, and magnetic-controlled microneedle robots] [22–28]. However, these therapeutic systems use a paracellular pathway or nonspecific passive transcellular pathway, resulting in a low peptide absorption rate and high variability in absorption. Additionally, some absorption enhancers require high concentrations to open tight junctions because of the buffering effect, which may induce GI-related adverse effects such as nausea, diarrhea, vomiting, and constipation [29].

Transporter-mediated endocytosis is a way of specifically targeting a transporter to facilitate cellular uptake of biological therapeutics [30–33]. Conventional targeted therapy typically relies on receptor-mediated endocytosis, which effectively targets specific receptors [34–38]. Although related signaling and endocytosis mechanisms have been reported, the transcellular pathway involved in exocytosis is unusual. In contrast, intestinal transporters are responsible for transporting nutrients and bile acids. Because they are highly expressed in the intestine, transporters such as peptide transporter 1, organic anion transporting polypeptides, and monocarboxylate transporter protein 1 are used for oral targeted delivery [39–41]. Above all, oral delivery systems using bile acids to target the apical sodium-dependent bile acid transporter (ASBT) have achieved significant improvements in the intestinal permeability of not only hydrophilic small-molecule anticancer drugs, but also macromolecules [42, 43]. ASBT-mediated endocytosis occurs when bile acids specifically bind to ASBT, inducing a transformational change in the occluded state of ASBT and the formation of ASBT vesicles for endocytosis [32, 44, 45]. This is promising for oral delivery of macromolecules because it avoids lysosomal degradation [46]. The detailed mechanism of ASBT-mediated endocytosis was clarified by investigation of ileal bile acid-binding protein and organic solute transporter α and β (OST_α/β_) [47]. Recent studies have shown that cellular absorption by ASBT-mediated endocytosis involves a multimodal mechanism, and that intracellular ASBT vesicles are trafficked by caveolae (specific lipid rafts) or a clathrin-independent pathway [48–50]. Targeting ASBT can increase the contact time, thus extending the interaction time between drugs and the intestinal epithelium, thereby enabling the utilization of transporter-mediated endocytosis and resulting in improved oral uptake of peptides [51].

**Scheme 1:**
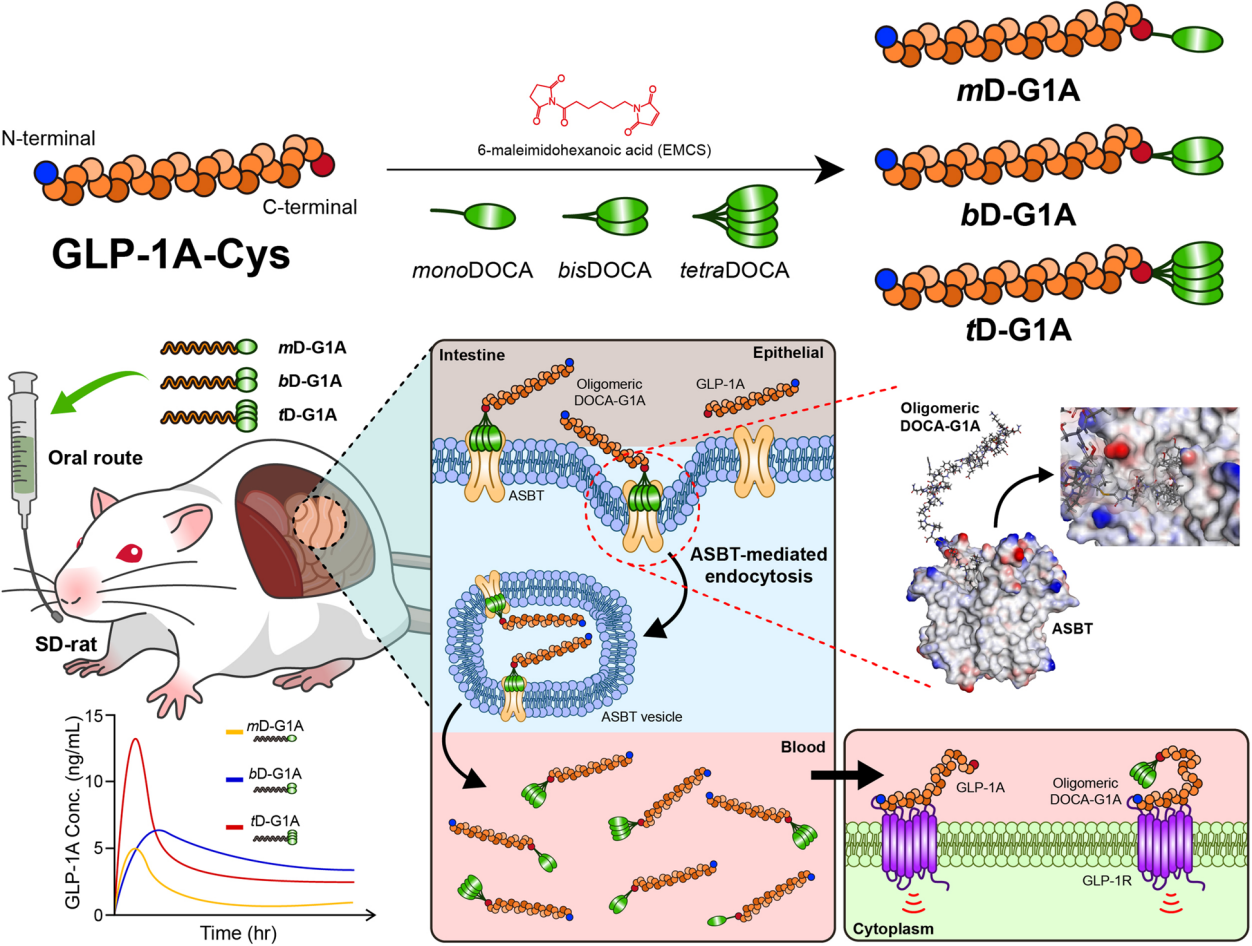
Scheme for developing oral chimeric GLP-1 agonists

This study was performed to develop an oral chimeric GLP-1A that can utilize ASBT-mediated endocytosis through conjugation with oligomeric bile acids (Scheme 1). Exenatide-based GLP-1A was selected as the GLP-1A model drug, and various chimeric oral GLP-1As were designed in silico to bind ASBT by conjugation with oligomeric bile acids. The binding affinity and dynamics between chimeric oral GLP-1A and ASBT were evaluated through molecular dynamics (MD) simulation in silico. To demonstrate proof of concept, GLP-1As conjugated with oligomeric bile acids were synthesized, and their insulinotropic effect was confirmed with pancreatic β islet cells. The in vitro ASBT binding cellular disposition was analyzed and a Caco-2 permeability assay was performed to evaluate cellular absorption. Additionally, in vivo pharmacokinetic (PK) parameters were evaluated to determine oral bioavailability, and pharmacodynamics were evaluated to determine the glucose-lowering effects in diabetic *db/db* mice. Thus, oral chimeric GLP-1As that utilize ASBT-mediated endocytosis were developed, starting from an in silico design and progressing to in vitro*/*in vivo PK–pharmacodynamic efficacies. This strategy can be expanded and applied to other existing GLP-1A sequences with high efficacy and stability, helping to reduce the time and cost of drug discovery to the non-clinical stage. This will in turn increase the development potential of oral GLP-1A candidates that can satisfy the growing oral GLP-1A market.

## Materials and methods

### Materials

Exenatide and GLP-1A-Cys were purchased from Anygen (Gwangju, Republic of Korea). Oligomeric deoxycholic acids (DOCAs) were synthesized from Mediplex (Seoul, Republic of Korea) with 4-methylmorpholine (4-MMP) (Sigma-Aldrich, St. Louis, MO, USA), dicyclohexylcarbodiimide (DCC) (Sigma-Aldrich), N-hydroxysuccinimide (NHS) (Sigma-Aldrich), lys(BOC)OMe and BOC-lysBOC-Osu, DOCA ethylenediamine [molecular weight (MW) of 434.7], *bis*DOCA ethylenediamine (MW of 937.4), and *tetra*DOCA ethylenediamine (MW of 1,942.9). For chemical conjugation of GLP-1A with oligomeric DOCAs, 6-maleimidohexanoic NHS, *N,N*-dimethylformamide (DMF), and triethylamine were purchased from Sigma-Aldrich. For in vitro experiments, Dulbecco’s modified Eagle’s medium (DMEM), phosphate-buffered saline (PBS), and Hank’s balanced salt solution (HBSS) were purchased from Corning Inc. (Corning, NY, USA). Krebs–Ringer bicarbonate buffer (KRBB) was purchased from Sigma-Aldrich. In addition, rabbit monoclonal anti-E cadherin (Abcam, Cambridge, UK), rabbit monoclonal anti-Rps20 (Abcam), anti-SLC10A2 rabbit polyclonal antibody (Bioss Antibodies, Woburn, MA, USA), and anti-rabbit immunoglobulin G–horseradish peroxidase secondary antibody (Sigma-Aldrich) were purchased for western blotting.

### Cell culture and animals

The Caco-2 cell line (human colorectal adenocarcinoma epithelial cells) and MDCK cell line (Madin–Darby canine kidney cells) were acquired from American Type Culture Collection (Manassas, VA, USA). The MDCK-ASBT cell line, which overexpresses ASBT, was transfected by a previously described method [45]. Complete culture DMEM medium was supplemented with 10% fetal bovine serum (Thermo Fisher Scientific, Waltham, MA, USA), 1% penicillin–streptomycin (Thermo Fisher Scientific), and 1% 1 × non-essential amino acid (Thermo Fisher Scientific). The cell culture environment was kept at 37 ℃ with 5% carbon dioxide and humidity, and the culture medium (DMEM) was refreshed every 2–3 days. When the cell confluence reached 80%, the cells were subcultured onto either a Transwell plate (Corning Inc.) for the permeability assay or a six-well plate (Corning Inc.) for the western blot.

The Institutional Animal Care and Use Committee guideline of Seoul National University approved all protocols for in vivo experiments involving animals (SNU-130801–3-1). Sprague–Dawley rats (male, 6 weeks old) and C57BLKs/J *db/db* mice (male, 7 weeks old) were purchased from Orient Bio Inc. (Seongnam-si, Republic of Korea) and Japan SLC, Inc. (Shizuoka, Japan), respectively. Prior to their use, all animals were stabilized for 1 week under controlled conditions (23 ℃ ± 2 ℃, 55% ± 10% humidity).

### In silico molecular docking

The molecular structure of oligomeric DOCA was drawn using ChemDraw Professional 20.1.1.125 (PerkinElmer, Waltham, MA, USA). Molecular docking was performed using AMDock (Assisted Molecular Docking) software with ASBT protein (PDB: 3ZUX) and CHARMM (Chemistry at Harvard Molecule Mechanics) force fields. The active site of the recorded ligand in the existing PDB was used as the binding site. From the 10 recorded poses, the best pose was selected and visualized using Discovery Studio 2022 software (BIOVIA, San Diego, CA, USA). Affinity and estimated Ki were calculated using AMDock software. Binding energy analysis was conducted using the “calculate binding energy protocol” with in situ ligand minimization and a generalized born with simple switching (GBSW) implicit solvent model.

### MD simulation of oligomeric DOCAs and oligomeric DOCA-G1As

MD simulation was conducted using the standard dynamic cascade protocol to observe the system in a constant-energy, constant-volume (NVE) ensemble. The GBSW implicit solvent model was used. The interaction energy between the oligomeric DOCA molecule and ASBT protein was calculated using a trajectory protocol. Trajectory analysis was performed during a 100-ps production step to ensure the conformation of the molecules and the strength of interactions in the system.

To assess the stability of oligomeric DOCA in relation to the ASBT protein, a docking process was used to generate the receptor-ligand pose for oligomeric DOCA. Subsequently, a 100-ps MD simulation was performed using the standard dynamic cascade protocol. The NVE ensemble and GBSW implicit solvent model were used during all processes. Conformational changes were recorded at specific time points after the 100-ps production step. The trajectory protocol was used to calculate the interaction energy between oligomeric DOCA and the binding cavity of the ASBT protein throughout the simulation.

The stable final pose obtained from the 100 ps MD simulation was used for conformational analysis of the ASBT binding cavity. To investigate the strength of the interaction of each DOCA motif of oligomeric DOCA-G1A, the following method was used to classify the poses of each DOCA motif. First, the centroid of each DOCA motif was calculated using the centroid feature in Discovery Studio software. The center of each determined motif was used to score the poses. According to the established rules, the center of DOCA motifs that were closer to the binding site were assigned higher scores. The interaction energy was then calculated for each motif in the selected poses. For example, in the case of *t*D-G1A, the interaction energy between each pose and the binding site was measured after ranking the four motifs.

### Preparation of oligomeric DOCA-G1As (*m*D-G1A, *b*D-G1A, and *t*D-G1A)

The synthesis of GLP-1A-conjugated oligomeric DOCA (oligomeric DOCA-G1As) involves three steps: synthesizing oligomeric DOCA-ethylenediamine, synthesizing oligomeric DOCA-linker, and conjugation of the oligomeric DOCA-linker to GLP-1A-Cys. For the first step, oligomeric DOCA was coupled with ethylenediamine to introduce linker 6-maleimidohexanoic NHS in accordance with a previously established method [43]. The conjugation of *mono*DOCA-ethylenediamine (*mono*DOCA) was achieved with activated DOCA using 4-MMP and ethylenediamine by a DCC/NHS coupling agent. *Bis*DOCA-ethylenediamine (*bis*DOCA) was conjugated with activated DOCA (16.85 mmol) using 4-MMP (16.85 mmol) and lys(BOC)OMe. After deprotection, ethylenediamine was coupled using a DCC/NHS reaction. To synthesize *tetra*DOCA-ethylenediamine (*tetra*DOCA), a lysine trimer was primarily prepared. First, H-lys(Boc)OMe-HCl (6.4 mmol) was dissolved in DMF, and 4-MMP (25.6 mmol) was added in a 1:4 ratio. Next, BOC-lysBOC-Osu (6.4 mmol) in DMF was added in a 1:1 ratio, and the mixture was reacted overnight under nitrogen purging. After deprotection, the same above-described reaction process was carried out with lys(Boc)OMe in DMF to produce the lysine trimer. *tetra*DOCA was synthesized by reacting activated DOCA and the lys-trimer for 3 days, and then purified using column chromatography (10% methanol/methyl chloroform). Ethylenediamine was then coupled using a DCC/NHS reaction after deprotection. For the second step, oligomeric DOCAs were conjugated to the linker 6-maleimidohexanoic NHS. Each oligomeric DOCA (1 eq.) was completely dissolved in DMF, and 6-maleimidohexanoic NHS (2 eq.) dissolved in DMF was added to the above solutions and mixed constantly. Triethylamine (2 eq.) was added dropwise, and the reaction was carried out for 1 h at room temperature. The resulting oligomeric DOCAs-linker was purified by reverse-phase high-performance liquid chromatography (RP-HPLC) using mobile solution A (water with 0.1% trifluoroacetic acid) and B (acetonitrile with 0.1% trifluoroacetic acid) at a flow rate of 1 mL/min on an ODS column (4.6 × 15 mm, 5 µm). Each peak corresponding to the oligomeric DOCAs-linker was purified and lyophilized for further steps. To conjugate the oligomeric DOCAs-linker with GLP-1A-Cys, GLP-1A-Cys (1 eq.) and oligomeric DOCAs-linker (10 eq.) were dissolved separately in DMF. The two solutions were mixed and reacted overnight at room temperature, then purified by HPLC system. GLP-1A-conjugated oligomeric DOCAs [*mono*DOCA-GLP-1A (*m*D-G1A), *bis*DOCA-GLP-1A (*b*D-G1A), and *tetra*DOCA-GLP-1A (*t*D-G1A)] were then concentrated, deionized using Centriprep (Sephadex G-25 desalting column), lyophilized, and identified by matrix-assisted laser desorption-time-of-flight mass spectrometry (MALDI-TOF MS) (Voyager DE-STR; Applied Biosystems, Waltham, MA, USA).

### Insulinotropic effect

The insulinotropic effects of oligomeric DOCA-G1As were compared by the static glucose-stimulated insulin secretion assay. Islet cells were isolated from Sprague–Dawley rats and suspended in KRBB containing low glucose (2.8 mM). The cells were seeded at a density of 20 IEG/insert in Millicell inserts and then pre-incubated for 1 h at 37 ℃ with 5% carbon dioxide and humidity. Samples [non-treated, exenatide (5 nM), GLP-1A-Cys (5 nM), *m*D-G1A (5 nM), *b*D-G1A (5 nM), and *t*D-G1A (5 nM)] were dissolved in KRBB, which contained low glucose (2.8 mM) and high glucose (28 mM). The inserts were first placed in low glucose-containing conjugates and incubated for 2 h. After incubation, the inserts were moved to high glucose-containing conjugates and incubated for the same duration. The insulin concentration in the low- and high-glucose groups was measured using a Rat/Mouse Insulin ELISA kit (Merck KGaA, Darmstadt, Germany). The stimulation index, which indicates the ability of insulin secretion to respond to glucose stimulation, was calculated by dividing the insulin concentration at high glucose by that at low glucose.

### Caco-2 permeability assay

The Caco-2 cell line was used to compare the cell permeability of oligomeric DOCA-G1As. Once the cells reached 70% confluence, the cells were subcultured and seeded into the apical side of a Transwell plate at a density of 10^4^ cells per well. The complete DMEM medium was exchanged every 3 days, with 400 μL for the apical side and 1 mL for the basolateral side; it was then incubated at 37 ℃ with 5% carbon dioxide and humidity until the transepithelial electrical resistance value reached (300 ohms). On the day of drug treatment, DMEM on the apical and basolateral sides was replaced with fresh HBSS. After 1 h, GLP-1A-Cys (1 μM), *m*D-G1A (1 μM), *b*D-G1A (1 μM), and *t*D-G1A (1 μM) dissolved in HBSS were applied to the apical side. Samples were taken from the basolateral side at predetermined times (1, 2, 3, and 6 h). The concentration of the treated materials was measured using an exendin-4 (*Heloderma suspectum*) EIA kit (Phoenix Pharmaceuticals, Burlingame, CA, USA). Permeability was calculated as follows:

$${P}_{app}=\frac{dQ}{dt} \times \frac{1}{A \times {C}_{0}}$$

where *P*_*app*_ represents the Caco-2 permeability, *dQ/dt* refers to the amount of drug in the basolateral side by time (mol/s), A refers to the area of the Transwell plate (cm^2^), and *C*_*0*_ refers to the initial concentration of drug on the apical side (mol/mL).

### ASBT-mediated transport of oligomeric DOCA-G1As

To investigate the involvement of an ASBT-mediated transcellular pathway in the permeation of oligomeric DOCA-G1A, a Caco-2 cell monolayer was prepared as previously described. The Caco-2 cell monolayer was preincubated with 0.1 mL of HBSS containing 3.2 μM actinomycin D (Act D) alone, 10 μM clofazimine (CFZ) alone, or a combination of both. Act D specifically inhibits ASBT, whereas CFZ is an inhibitor of OST_α/β_ transporters. The cells were incubated with the inhibitors for 30 min at 37 °C. After preincubation, the apical chamber was filled with 0.1 mL of oligomeric DOCA-G1A in HBSS (equivalent to 100 μg/mL exenatide), along with the corresponding inhibitor. The solution in the basolateral chamber was replaced with 0.6 mL of fresh HBSS. The cells were then incubated at 37 °C for 1, 2, 3, or 6 h. At the predetermined time points, 200 µL of sample solution were withdrawn from the basolateral chamber of each well, and an equal quantity of fresh HBSS was added to maintain consistent volume. The amount of oligomeric DOCA-G1A that permeated across the Caco-2 cell monolayer was quantified using the exendin-4 EIA kit (Phoenix Pharmaceuticals). *P*_*app*_ was determined by calculating the linear slope of the cumulative permeated amount of oligomeric DOCA-G1A plotted as a function of time, using the equation mentioned in the previous section. The relative *P*_*app*_ for each oligomeric DOCA-G1A was calculated by comparison with the *P*_*app*_ obtained in the absence of inhibitor.

### Western blot analysis for ASBT-mediated endocytosis

To investigate the mechanism of transporter-mediated endocytosis of oligomeric DOCA-G1As, the distribution of ASBT on MDCK-ASBT was identified. The MDCK-ASBT cell line, which overexpresses ASBT, was cultured with complete DMEM medium until it reached 80% confluence. On the day of drug treatment, the cells were moved to HBSS and pre-incubated at 37 ℃ for 1 h. *t*D-G1A was prepared at a concentration of 1 μM and applied for 1 h at 37 ℃. After incubation, the membrane protein fraction and cytosol protein fraction were separated using Mem-PER™ (Thermo Fisher Scientific) in accordance with the manufacturer’s protocol. Briefly, the cells were washed three times with PBS and scraped off the surface of the plate with PBS containing protease inhibitor cocktail (GenDEPOT, Baker, TX, USA). The suspended cells were centrifuged at 300 × *g* for 5 min, and the supernatant was discarded. The pellet was treated with permeabilization buffer and vortexed for 10 min at 4 ℃ with consistent mixing, followed by centrifugation at 15,000 × *g* for 45 min at 4 ℃. The supernatant was collected as the cytosolic fraction. Solubilization buffer was then added to the remaining pellet, which was vortexed at 4 ℃ for 60 min with consistent mixing, followed by centrifugation at 15,000 × *g* for 15 min at 4 ℃. The supernatant was collected as the membrane fraction. The protein concentration was measured using a bicinchoninic acid protein assay kit (Thermo Fisher Scientific). The distribution of ASBT was confirmed by western blotting with the membrane marker anti-E cadherin and cytosolic marker anti-RPS20. A total of 50 μg of protein was loaded onto 12% Tris–glycine gel (Bio-Rad Laboratories, Hercules, CA, USA), followed by electrophoresis and membrane transfer. The primary antibodies, including anti-SLC10A2, anti-E cadherin, and anti-RPS20, were used to capture the target proteins. Finally, horseradish peroxidase-conjugated secondary antibody was applied, and the analysis was performed using a LAS 4000 imaging system (GE Healthcare, Chicago, IL, USA).

### PK study

Male Sprague–Dawley rats (6 weeks old, 180–200 g) were used to evaluate the oral absorption of oligomeric DOCA-G1As. Prior to drug treatment, the rats fasted overnight for oral administration. On the drug treatment day, 3% sodium bicarbonate was administered as an antacid via oral gavage 10 min before drug administration. GLP-1A-Cys was administered intravenously (10 μg/kg) and orally (100 μg/kg). Also, oligomeric DOCA-G1As (*m*D-G1A, *b*D-G1A, and *t*D-G1A) were administered orally (100 μg/kg). At predetermined times (15 min, 30 min, 1 h, 1.5 h, 2 h, 4 h, and 6 h), whole blood was collected from the jugular vein, and the plasma was separated by centrifugation at 4500 × *g* and 4 ℃ for 15 min. The plasma drug concentration was analyzed using an exendin-4 (*Heloderma suspectum*) EIA kit (Phoenix Pharmaceuticals). Non-compartmental analysis was used to calculate the PK parameters.

### Intraperitoneal glucose tolerance test (IPGTT) in diabetic (db/db) mouse model

To evaluate glucose tolerance and the hypoglycemic effects of oligomeric DOCA-G1As, an IPGTT was performed using a diabetic mouse model (C57BLKs/J *db*/*db*). On the day of drug treatment, the mice were fasted for 5 h and administered 3% sodium bicarbonate as an antacid. Exenatide (5 µg/kg, subcutaneous), *b*D-G1A (50 μg/kg, oral), or *t*D-G1A (50 μg/kg, oral) was administered to the mice. After a 15-min interval, blood samples were collected from the tail vein to measure initial glucose levels (0 h). This collection was followed by intraperitoneal injection of glucose (2 g/kg). Subsequently, blood samples were collected, and glucose levels were measured at predetermined time points (0.25, 0.5, 1, 1.5, 2, 2.5, 3, 3.5, 4, and 5 h) after glucose injection. Furthermore, the hypoglycemic effects of *b*D-G1A and *t*D-G1A were assessed in *db*/*db* mice with initial glucose levels of 500–600 mg/dL. For this assay, mice were fasted for 5 h and administered 3% sodium bicarbonate; they subsequently received exenatide (5 µg/kg, subcutaneous), GLP-1A-Cys (20 μg/kg, oral), *b*D-G1A (20 μg/kg, oral), or *t*D-G1A (20 μg/kg, oral). Glucose levels were measured using a glucometer for up to 10 h.

### Statistical analysis

All statistical analyses and graphics were plotted using Prism 9.0 (GraphPad Software, San Diego, CA, USA). Non-compartmental analysis was performed to calculate PK parameters using WinNonlin 5.0.1 software (Pharsight Corporation, Sunnyvale, CA, USA). A *t*-test or one-way analysis of variance was performed to compare groups. In all cases, the *p*-value was two-tailed and indicated as follows: ^*^p < 0.05, ^**^p < 0.01, and ^***^p < 0.001. A *p*-value of < 0.05 was considered statistically significant.

## Results

### In silico ASBT binding analysis of oligomeric DOCAs

**Fig. 1:**
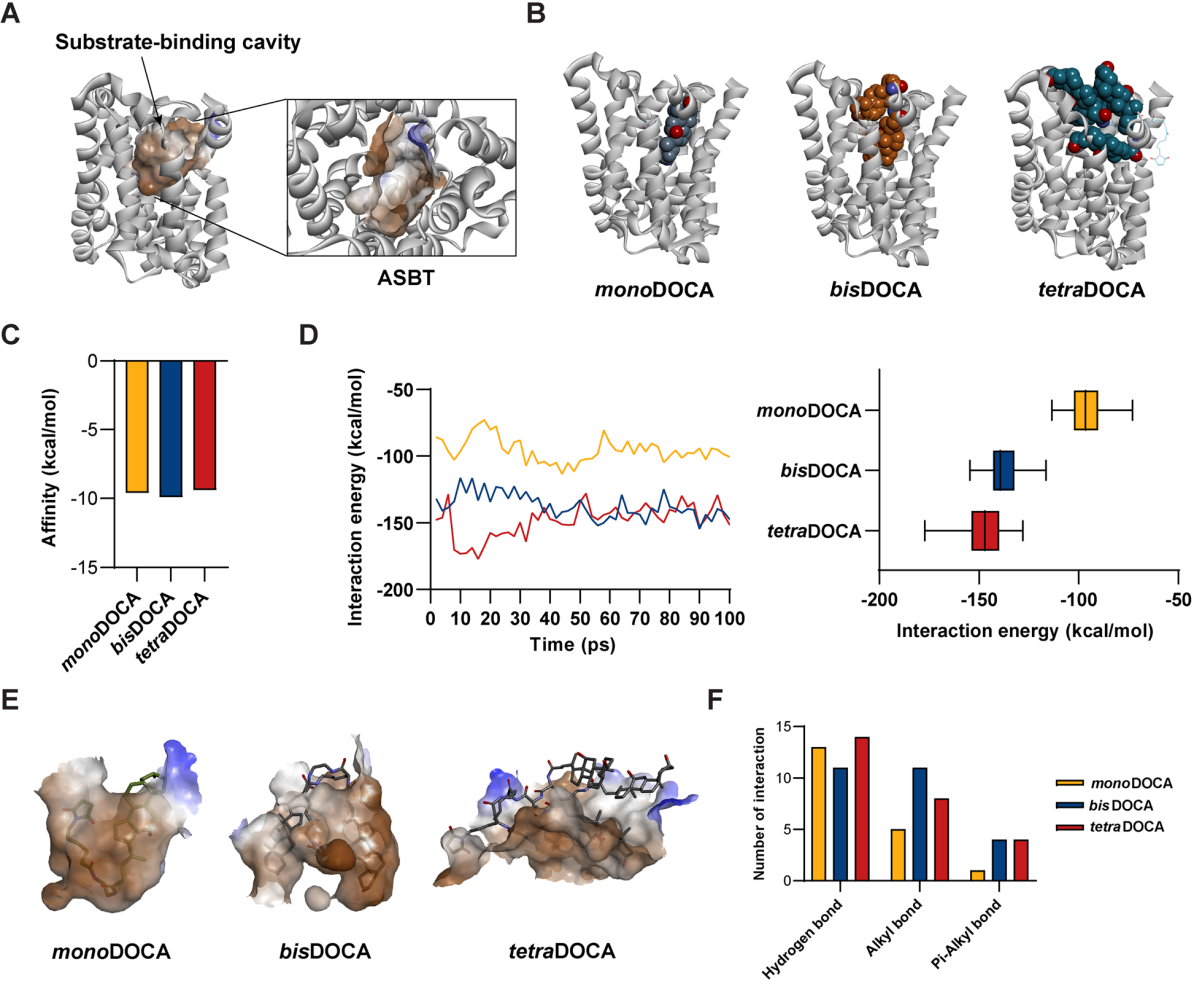
In silico molecular docking analysis and molecular dynamics simulation between oligomer DOCAs and apical sodium bile acid transporter (ASBT). **A** Substrate-binding cavity colored in back born structure of ASBT and **B** docking simulation of oligomeric DOCAs (*mono*DOCA, *bis*DOCA, and *tetra*DOCA) to the pore of ASBT. **C** Binding affinities of oligomeric DOCAs to ASBT. After MD simulation, **D** the interaction energy over the time range of 0 to 100 ps during MD simulation and average interaction energy. **E** Stabilized binding poses of oligomeric DOCAs, and **F** categories and counts of interactions between oligomeric DOCAs and ASBT

In silico molecular docking analysis was performed to investigate the potential inhibitory effect of oligomeric DOCA on the interaction with ASBT protein. The binding site of ASBT protein, which is known to contain hydrophobic cavities favorable for binding with hydrophobic molecules (Fig. 1A), was visualized based on its hydrophobicity. Molecular docking was performed for *mono*DOCA, *bis*DOCA, and *tetra*DOCA in the binding site (Fig. 1B). *mono*DOCA bound adequately to the hydrophobic cavity of ASBT, but occupied a smaller volume than the binding area. However, *bis*DOCA and *tetra*DOCA bound stably to the binding site of ASBT and occupied a larger volume than *mono*DOCA. In the quantitative analysis of binding affinity, *bis*DOCA showed the highest binding affinity, with a value of − 9.90 kcal/mol, followed by *mono*DOCA with a lower affinity of − 9.60 kcal/mol. Interestingly, *tetra*DOCA showed the lowest affinity, at − 9.40 kcal/mol (Fig. 1C). All interactions were strong, with binding affinities of <  − 9 kcal/mol, indicating that even with an increase in DOCA motifs, the binding affinity was not significantly affected or even increased.

Further analysis using the GBSW (Generalized born with a sample switching) method to apply virtual solvent effects and measure the binding energy was performed to gain a deeper understanding of the interaction between oligomeric DOCA and ASBT protein. The binding energy of *tetra*DOCA was the lowest, at − 47.44 kcal/mol, followed by *bis*DOCA with a binding energy of − 41.21 kcal/mol and *mono*DOCA with a binding energy of − 35.85 kcal/mol (Fig. S1). The estimated Ki values, as calculated by AMDock software, revealed that *bis*DOCA exhibited the highest inhibitory potency with a Ki value of 55.37 nM, while *tetra*DOCA showed a lower inhibitory potency of 128.75 nM compared with *mono*DOCA at 91.87 nM (Fig. S1).

### Molecular dynamic (MD) simulation of oligomeric DOCAs

MD simulations were performed for the receptor-ligand complex of oligomeric DOCA and ASBT protein over a duration of 100-ps. The aim was to investigate the interaction energy and evaluate the binding sites and strength of interaction between *mono*DOCA, *bis*DOCA, or *tetra*DOCA and ASBT protein. The interaction energy was analyzed to understand the dynamics of interactions during the 100-ps MD simulation. The results demonstrated that *mono*DOCA exhibited the lowest energy at all time points, indicating the strongest interaction. *Bis*DOCA and *tetra*DOCA had similar interaction energy levels throughout the simulation (Fig. 1D). *Tetra*DOCA initially exhibited the lowest energy, indicating a strong interaction up to 38-ps; thereafter, its interaction energy trend aligned with the energy of *bis*DOCA (Fig. 1D). From 0 to 100-ps, *tetra*DOCA displayed the lowest average interaction energy value of − 148.27 kcal/mol, indicating the strongest interaction. *Bis*DOCA exhibited interaction energy of − 137.45 kcal/mol, whereas *mono*DOCA exhibited interaction energy of − 95.62 kcal/mol (Fig. 1D). After completion of the MD simulation, the types and strengths of interactions between stable poses of oligomeric DOCA and ASBT protein were evaluated (Fig. 1E). *Mono*DOCA consistently displayed the lowest levels of all interactions, except for hydrogen bonds. This finding can be attributed to the increased hydrophobic motif of DOCA in *bis*DOCA and *tetra*DOCA, which increases alkyl bonds because of enhanced molecule hydrophobicity (Fig. S2). Notably, although both *bis*DOCA and *tetra*DOCA exhibited higher levels of hydrophobic interactions compared with *mono*DOCA, the increase in hydrophobic interactions was weaker in *tetra*DOCA than in the DOCA motif. Instead, *bis*DOCA demonstrated an increase in alkyl bonds (Fig. 1F). Overall, the results obtained from the 100-ps MD simulation indicated that *bis*DOCA and *mono*DOCA had the highest and lowest number of interactions, respectively, across all interaction types.

### MD simulation of oligomeric DOCA-G1As (*m*D-G1A, *b*D-G1A, and *t*D-G1A)

**Fig. 2:**
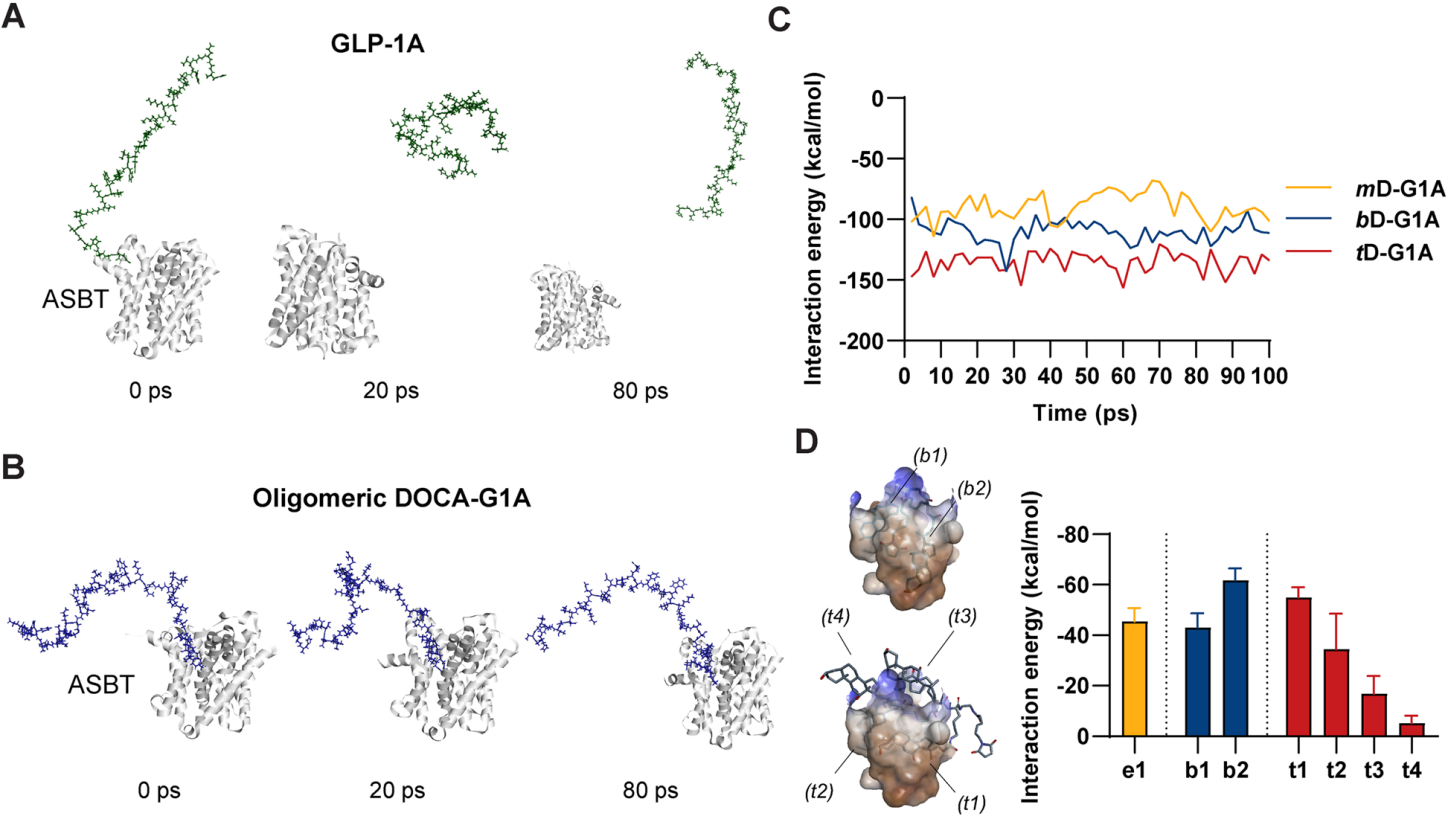
MD simulations of GLP-1A and oligomeric DOCA-G1As. **A** Conformations of ASBT and GLP-1A were recorded at specific time intervals (baseline to 80 ps) during the MD simulation. **B** Recorded conformations of ASBT and oligomeric DOCA-G1As. **C** Continuous change in interaction energy from 0 to 100 ps between ASBT and oligomeric DOCA-G1As. **D** Interaction energy between ASBT and each DOCA motif in oligomeric DOCA

The interaction between oligomeric DOCA-G1A and ASBT protein was evaluated by docking oligomeric DOCA-G1A peptides to the DOCA motif of ASBT protein. Subsequently, a 100 ps MD simulation was performed using the GBSW implicit solvent model under the constant-temperature, constant-pressure (NTP) ensemble. The MD simulation results showed that without oligomeric DOCAs, no significant interaction occurred between GLP-1A peptide and ASBT protein, and dissociation of GLA-1A was observed at 20 ps (Fig. 2A). However, when GLP-1A peptide was conjugated with oligomeric DOCAs, it remained stably bound to the three-dimensional structure of ASBT protein without dissociation during the 100 ps MD simulation (Figs. 2B and S3). Compared with the interaction energies of oligomeric DOCA-G1As, the overall interactions were higher than when GLP-1A peptide was not bound. However, the overall trend was similar to the results of oligomeric DOCAs, with *t*D-G1A showing the lowest interaction energy and the strongest interaction (Fig. 2C).

The conformation of each DOCA motif in the docked structures of *m*D-G1A, *b*D-G1A, and *t*D-G1A was investigated to understand their interactions with the ASBT binding site and determine which motif position contributed the most to the interaction. The results showed that *m*D-G1A with one DOCA motif (e1) exhibited an interaction energy of − 47 kcal/mol (Fig. S4). *b*D-G1A with two DOCA motifs showed interaction energies of − 60 kcal/mole (b2) and − 45 kcal/mole (b1). By contrast, *t*D-G1A with four DOCA motifs showed interaction energies of − 58 kcal/mol (t1) and − 37 kcal/mole (t2), while (t3) and (t4) motifs showed interaction energies below − 20 kcal/mol (Fig. 2D).

### Synthesis of oligomeric DOCA-G1As

**Fig. 3:**
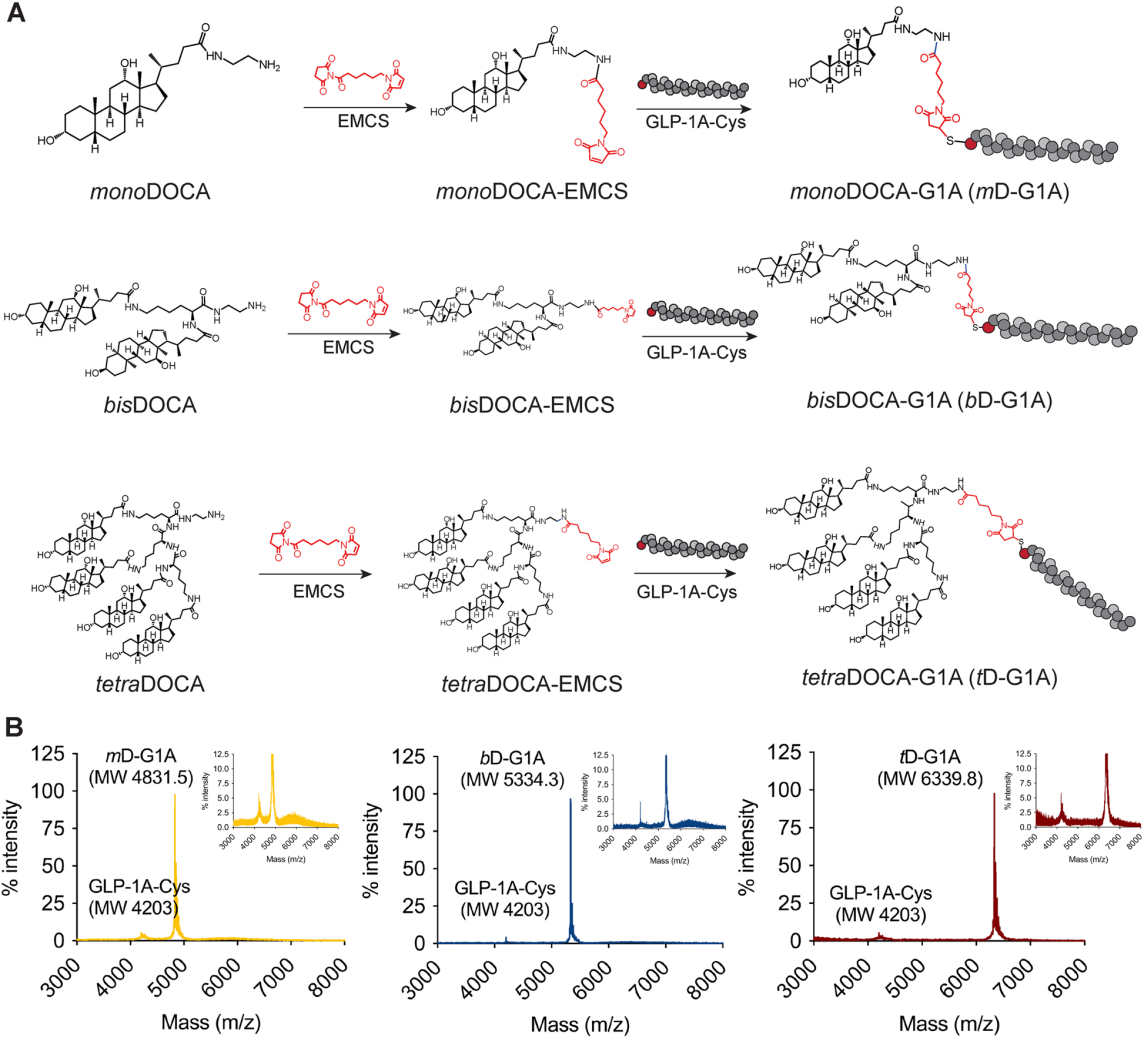
Synthesis of oligomeric DOCA-G1As. **A** Schematic synthesis steps for *mono*DOCA-G1As (*m*D-G1A), *bis*DOCA-G1A (*b*D-G1A), and *tetra*DOCA-G1A (*t*D-G1A); conjugated oligomeric DOCAs to GLP-1A-Cys with 6-maleimidohexanoic NHS (EMCS); and **B** MALDI-TOF MS identification

The oligomeric DOCA-G1As were synthesized using a maleimide reaction with an initial in silico design (Fig. 3A). The purified oligomeric DOCAs, including *mono*DOCA (MW: 434.7 Da), *bis*DOCA (MW: 937.4 Da), and *tetra*DOCA (MW: 1942.9 Da), were initially identified using RP-HPLC and ^1^H-NMR. The NMR data of the oligomeric DOCAs revealed new N–H peaks (Fig. S5). Subsequently, the oligomeric DOCAs were conjugated to the 6-maleimidohexanoic NHS (EMCS) linker, and the conjugates were then purified using RP-HPLC preparative isolation. The NMR results displayed new characteristic peaks (Fig. S6) and the corresponding mass were identified at 650.5 m*/z* (*m*D-G1A + Na), 1153.6 m*/z* (*b*D-G1A + Na), and 2159.9 m*/z* (*t*D-G1A + Na) (Fig. S7). Finally, the oligomeric DOCA-EMCS conjugates were reacted with the thiol group of GLP-1A-Cys to generate the oligomeric DOCA G1As. All materials were purified by HPLC to achieve > 90% purity. However, the yields of the final products from the initial 5 mg of GLP-1A-Cys were 83.3%, 89.1%, and 70.8% for *m*D-G1A, *b*D-G1A, and *t*D-G1A, respectively. These findings indicate that *t*D-G1A exhibited the lowest yield, suggesting that higher MW oligomeric DOCAs resulted in lower conjugation yields. The purified products, *m*D-G1A (MW: 4831.5 Da), *b*D-G1A (MW: 5334.3 Da), and *t*D-G1A (MW: 6339.8 Da), were confirmed through MALDI-TOF MS analysis (Fig. 3B), validating the synthesis approach.

### In vitro cellular uptakes and insulinotropic effect

**Fig. 4:**
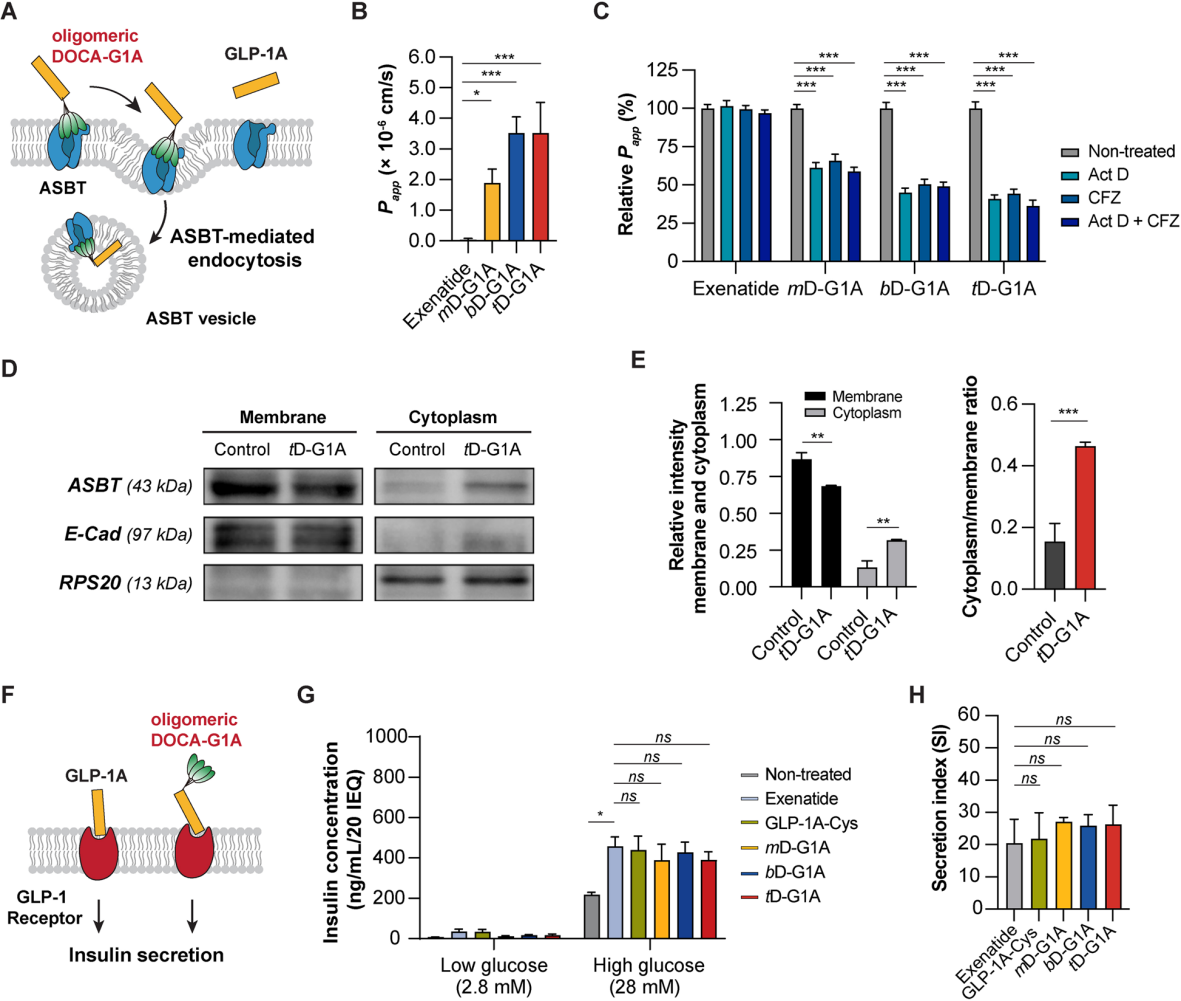
In vitro efficacy of chimeric GLP-1A for ASBT-mediated endocytosis and insulinotropic effect. **A** Scheme of the cellular uptake mechanism for GLP-1A and oligomeric DOCA-G1As utilizing ASBT-mediated endocytosis, leading to the formation of ASBT vesicles in cytosol. **B** Cellular permeability in Caco-2 cells after treatment with 1 μM of exenatide, *m*D-G1A, *b*D-G1A, and *t*D-G1A (*n* = 3; data are presented as means ± standard deviations). ^*^p < 0.05 and ^***^p < 0.001 compared with exenatide. **C** Relative *P*_*app*_ of oligomeric DOCA-G1A in the presence of actinomycin D (Act D) alone, clofazimine (CFZ) alone, or both (Act D + CFZ) (*n* = 4; data are presented as means ± standard deviations). ^***^p < 0.001 compared with relative *P*_*app*_ of the non-treated group. **D** ASBT distribution from the membrane and cytoplasm after administration of 1 μM of *t*D-G1A. **E** Relative signal intensity of ASBT in membrane and cytoplasm. ASBT quantification was conducted using ImageJ software (*n* = 3; data are presented as means ± standard deviations). ^**^p < 0.01 and ^***^p < 0.001 compared with the control. **F** Scheme of GLP-1R binding of oligomeric DOCA-G1A. **G** Insulin secretion (*n* = 4; data are presented as means ± standard deviations) by islet β cells in low-glucose (2.8 mM) and high-glucose (28 mM) conditions after drug treatment [non-treated; exenatide, 5 nM (control); GLP-1A-Cys, 5 nM; *m*D-G1A, 5 nM; *b*D-G1A, 5 nM; *t*D-G1A, 5 nM] and **H** the secretion index. ^*^p < 0.05, ^**^p < 0.01, and ^***^p < 0.001 compared with exenatide

To assess the cellular permeability and uptake mechanism of oligomeric DOCA-G1As for in vivo oral absorption, we investigated their interaction with ASBT and their transport across the intestinal epithelial membrane. We found that oligomeric DOCA-G1As strongly bind to ASBT, resulting in the formation of ASBT vesicles through ASBT-mediated endocytosis. These vesicles are then internalized and reach the cytosol (Fig. 4A). The effectiveness of oligomeric DOCA-G1As with increasing cellular permeability was assessed using the Caco-2 cell line, based on a previously established method. The results demonstrated that when exenatide was applied to the apical side of the Caco-2 cell monolayer, it did not effectively permeate to the basolateral side, as indicated by its low *P*_*app*_ value (0.04 ± 0.04, × 10^–6^ cm/s). In contrast, *m*D-G1A exhibited a significantly higher *P*_*app*_ value (1.89 ± 0.45, × 10^–6^ cm/s). Notably, *b*D-G1A (3.52 ± 0.52, × 10^–6^ cm/s) and *t*D-G1A (3.52 ± 1.00, × 10^–6^ cm/s) demonstrated similar *P*_*app*_ values, which were approximately 90.2-fold and 90.1-fold higher than the *P*_*app*_ value of exenatide, respectively (Fig. 4B). To confirm the involvement of ASBT-facilitated transport of oligomeric DOCA-G1As across the epithelial membrane in the GI tract, the Caco-2 cell permeability of each oligomeric DOCA-G1A was analyzed in the presence or absence of specific inhibitors for ASBT (Act D) and OST_α/β_ (CFZ). Treatment with Act D alone resulted in a significant decrease in the *P*_*app*_ value of *m*D-G1A by 38.8%, compared with the untreated control (Fig. 4C). Upon conjugation of *bis*DOCA or *tetra*DOCA to the GLP-1A peptide, their *P*_*app*_ values were further reduced by 1.42-fold and 1.52-fold, respectively, compared with *m*D-G1A after Act D treatment. Similarly, OST_α/β_ inhibition by CFZ led to decreases in the *P*_*app*_ values of *m*D-G1A, *b*D-G1A, and *t*D-G1A by 34.1%, 49.5%, and 55.6%, respectively, compared with their corresponding controls without inhibitor (Fig. 4C). Furthermore, combined treatment with Act D and CFZ resulted in a greater reduction in the *P*_*app*_ value of *m*D-G1A (41.2% decrease compared with the control in the absence of inhibitors), as well as reductions of 50.9% for *b*D-G1A and 63.6% for *t*D-G1A (Fig. 4C). In contrast, exenatide exhibited minimal permeation across the Caco-2 cell monolayer, regardless of the presence of inhibitors (Fig. 4C).

Furthermore, Western blot analysis was performed after cells had been treated with *t*D-G1A for 1 h; the results showed the presence of ASBT bands in both membrane and cytosol fractions (Fig. 4D). To quantify the expression of ASBT in the membrane and cytoplasm, we used ImageJ software. The relative intensity of ASBT in the cytoplasm fraction significantly increased from 0.13 ± 0.05 to 0.32 ± 0.01 after *t*D-G1A treatment. Moreover, the cytoplasm-to-membrane ratio was 3.0-fold higher in the *t*D-G1A-treated group than in the control group (Fig. 4E). These observations indicate that *t*D-G1A induces ASBT translocation from the membrane to the cytosol.

The cumulative results indicate that the permeation of oligomeric DOCAs-G1A involves ASBT-facilitated uptake. The interaction between oligomeric DOCA and ASBT allows the conjugate to effectively cross ileal enterocytes. Intracellular trafficking may also be facilitated by intestinal bile acid binding protein, guiding the conjugate within the cytosol. Finally, the conjugate escapes through the basolateral membrane via OST_α/β_, ensuring its transcellular transport.

To evaluate their insulinotropic effects (Fig. 4F), oligomeric DOCA-G1As were compared with GLP-1A-Cys and exenatide on islet β cells under low-glucose (2.8 mM) and high-glucose (28 mM) conditions. The insulin secretion in all drug-treated groups under low-glucose conditions was not significantly different from that in the control group treated only with HBSS. However, under high-glucose conditions, exenatide secreted insulin at a rate 211.2% higher than the non-treated group. GLP-1A-Cys, which was not conjugated with any oligomeric DOCAs, showed a level of insulin secretion (202.4%) similar to that of exenatide. The oligomeric DOCA-G1As (*m*D-G1A, *b*D-G1A, and *t*D-G1A) did not significantly differ from exenatide in terms of insulin secretion (179.4%, 197.2%, and 179.7%, respectively) (Fig. 4G). Furthermore, the secretion index, which represents the absolute insulinotropic effect calculated by dividing the insulin level under high-glucose conditions by that under low-glucose conditions, was evaluated. The results indicated that the insulin secretion ability of oligomeric DOCA-G1As was not significantly different from that of GLP-1A-Cys and exenatide (Fig. 4H).

### In vivo oral absorption in rats

**Fig. 5:**
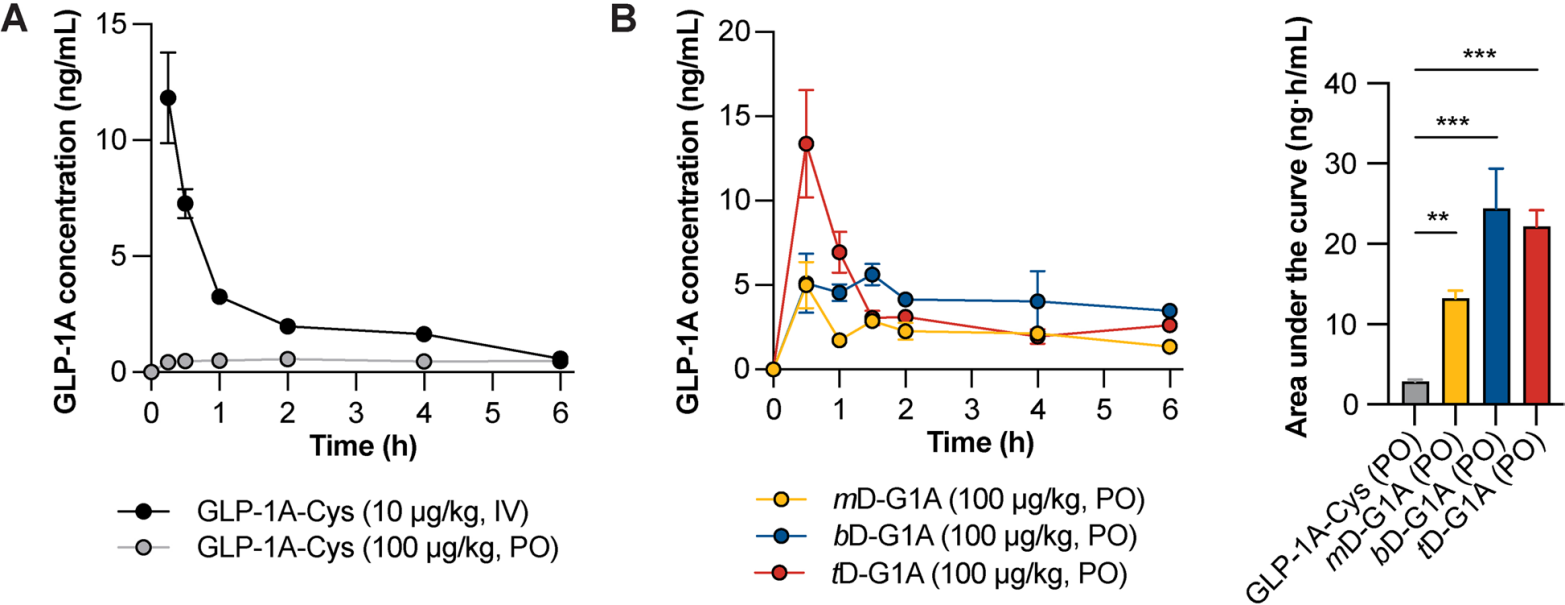
In vivo oral absorption of GLP-1A-Cys and oligomeric DOCA-G1As. **A** Time course of GLP-1A concentration after treatment with intravenous GLP-1A-Cys (10 μg/kg, *n* = 4), oral GLP-1A-Cys (100 μg/kg, *n* = 4), and **B** oral oligomeric DOCA-G1As (100 μg/kg, *n* = 4). Data are presented as mean ± standard deviation. ^**^p < 0.01 and ^***^p < 0.001 compared with GLP-1A-Cys

A PK study was conducted in Sprague–Dawley rats to evaluate the in vivo absorption of oligomeric DOCA-G1A. The time course of the plasma GLP-1A concentration was analyzed. PK parameters and absolute bioavailability were calculated based on GLP-1A-Cys. Intravenously administered GLP-1A-Cys showed a half-life of 2.1 ± 0.2 h. When administered orally, it showed low absorption and the oral bioavailability was 1.04% (Fig. 5A and Table 1). In case of oligomeric DOCA-G1As, *m*D-G1A and *t*D-G1A showed T_max_ of 0.5 h, while *b*D-G1A showed a flip-flop profile after 1.5 h of T_max_ (Fig. 5B). According to their areas under the curve, *b*D-G1A and *t*D-G1A showed higher absorption rates than *m*D-G1A. Consequently, the oral bioavailability of oligomeric DOCA-G1As was calculated as 4.7%, 8.6%, and 7.6% for *m*D-G1A, *b*D-G1A, and *t*D-G1A, respectively (Table 1).

**Table 1:** Pharmacokinetic parameters of GLP-1A-Cys and oligomeric DOCA-G1As

	GLP-1A-Cys	GLP-1A-Cys	*m*D-G1A	*b*D-G1A	*t*D-G1A
Administration route	IV	PO	PO	PO	PO
Dose (μg/kg)	10	100	100	100	100
T_max_ (h)		2.0	0.5	1.5	0.5
C_max_ (ng/mL)		0.56 ± 0.08	5.01 ± 2.37	7.63 ± 0.90	13.39 ± 5.54
AUC_0-last_ (ng·h/mL)	17.38 ± 0.70	2.88 ± 0.21	13.27 ± 0.91	24.43 ± 4.97	22.14 ± 2.03
MRT (h)	11.2 ± 1.7	27.3 ± 22.8	7.6 ± 3.8	7.9 ± 1.3	6.6 ± 2.6
BA (%)	100	1.0 ± 0.1	4.7 ± 0.3	8.6 ± 1.7	7.6 ± 0.7

*Abbreviations: IV* intravenous, *PO* per oral, *T*_*max*_ time of maximum concentration, *C*_*max*_ maximum concentration, *AUC* area under the curve, *MRT* mean residence time, *BA* bioavailability compared with IV route

Data are presented as mean ± standard deviation (*n* = 4 for each group). T_max_ represents the median

### Hypoglycemic effect of oligomeric DOCA-G1As

**Fig. 6:**
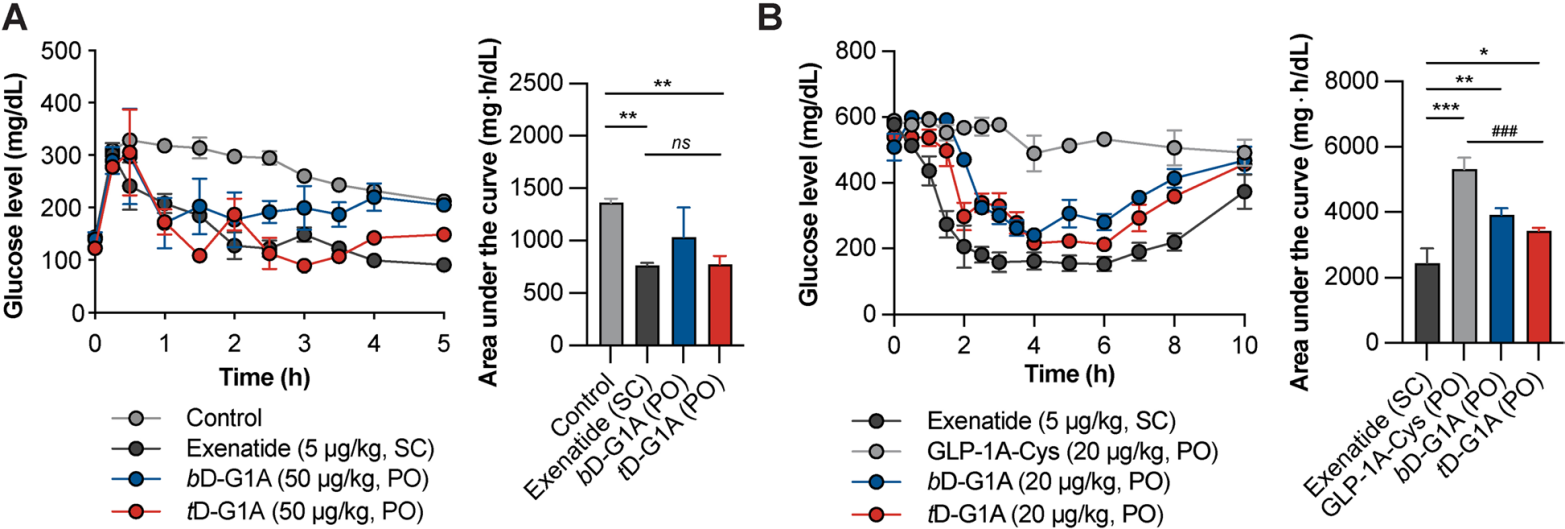
Hypoglycemic effect of oligomeric DOCA-G1As in *db/db* mice. **A** IPGTT after treatment with control (saline, oral, *n* = 4), exenatide (5 µg/kg, subcutaneous, *n* = 4), *b*D-G1A (50 μg/kg, oral, *n* = 4), and *t*D-G1A (50 μg/kg, oral, *n* = 4) following glucose (2 g/kg) injection. Data are presented as mean ± standard deviation. ^**^p < 0.01 compared with the control. **B** Time course of changes in blood glucose levels of *db/db* mice displaying a high fasting glucose level after treatment with exenatide (5 µg/kg, subcutaneous, *n* = 4), GLP-1A-Cys (20 μg/kg, oral, *n* = 4), *b*D-G1A (20 μg/kg, oral, *n* = 4), and *t*D-G1A (20 μg/kg, oral, *n* = 4). Data are presented as means ± standard deviations. ^*^p < 0.05, ^**^p < 0.01, and ^***^p < 0.001 compared with exenatide. ^###^*p* < 0.001 compared with GLP-1A-Cys

An IPGTT was conducted in *db*/*db* mice to compare the glucose-lowering effect of oral oligomeric DOCA-G1As (*b*D-G1A and *t*D-G1A). Considering that oral glucose can interfere with the absorption of orally administered drugs, exenatide (5 µg/kg, subcutaneous), *b*D-G1A (50 μg/kg, oral), and *t*D-G1A (50 μg/kg, oral) were administered prior to the intraperitoneal injection of glucose (2 g/kg). All treated groups exhibited hypoglycemic effects within 1 h. Both *b*D-G1A and *t*D-G1A reduced the minimum glucose level to 170.3 mg/dL at 1 h and 89.7 mg/dL at 3 h (Fig. 6A). Notably, the *t*D-G1A group maintained fasting glucose levels < 200 mg/dL for up to 5 h, similar to the effects observed with exenatide. However, the *b*D-G1A group displayed fasting glucose levels > 200 mg/dL beginning at 4 h, similar to the control group. Area under the curve of the glucose levels were reduced by 47.1 ± 5.9% with *t*D-G1A and 44.2 ± 1.8% with exenatide, while *b*D-G1A caused a reduction of 24.1 ± 20.7%. In *db*/*db* mice with induced diabetes (glucose levels > 500 mg/dL), the changes in glucose levels in response to *b*D-G1A and *t*D-G1A were measured in comparison with oral GLP-1A-Cys (negative control) and subcutaneous exenatide (positive control) (Fig. 6B). Both oral oligomeric DOCA-G1As effectively lowered the glucose levels to 200 mg/dL after 2 h, and this decrease was sustained for up to 6 h. After 10 h, the glucose levels returned to levels observed in the negative control group. Area under the curve analysis revealed that *b*D-G1A, *t*D-G1A, and exenatide (positive control) reduced the glucose levels by 26.4 ± 3.9%, 35.5 ± 1.7%, and 54.3 ± 8.5%, respectively.

## Discussion

GLP-1A, a highly effective therapeutic peptide for diabetes, exerts its effects through various mechanisms [52]. However, the route of administration is limited to injection because of its large MW and high hydrophilicity, making it difficult for GLP-1A to penetrate hydrophobic membranes and resulting in low cellular permeability. Cellular penetration may occur by two pathways: the paracellular and transcellular pathways. The paracellular pathway is limited because the available surface area is < 1% of the entire intestine, making it difficult to increase oral bioavailability. Additionally, use of the transcellular pathway with an absorption enhancer can induce high absorption variability and cellular toxicity because it disrupts the cell membrane. However, transporter-mediated endocytosis enables oral GLP-1A to utilize transporters, thus increasing the cellular permeability of the peptide [30]. Therefore, we designed an oral chimeric GLP-1A that not only activates GLP-1R but also utilizes transporter-mediated endocytosis for oral delivery (Scheme 1). Exenatide was selected as the model drug for oral GLP-1A because it shares 53% sequence similarity with GLP-1 (7–37) and exhibits high binding affinity to GLP-1R (K_d_ = 136 pM) [53]. A targeting moiety to ASBT was introduced in a specific sequence of exenatide that does not affect binding or activity. In the case of liraglutide and semaglutide, lipidation was introduced into lysine at position 26 for albumin binding, but they showed lower GLP-1R binding (liraglutide K_d_ = 0.11 nM, semaglutide K_d_ = 0.38 nM) for a long half-life [54]. We targeted the *c*-terminal region rather than the *n*-terminal region as the target sequence for GLP-1R binding, while maintaining the sequence of GLP-1A as much as possible to avoid affecting binding to GLP-1R. To conjugate an oral absorption moiety specific to the *c*-terminal, GLP-1A-Cys with a thiol group was used instead of serine at position 39. The intracellular cyclic adenosine monophosphate production of GLP-1A-Cys (E_max_: 133.2 nM, EC_50_: 2.9 nM) in PC12 cells was similar to that of exenatide (E_max_: 129.4 nM, EC_50_: 2.3 nM) [55]. Bile acids are commonly used as drug carriers because they bind and transport via ASBT. When oligomers were made using bile acids, they showed higher binding affinity with ASBT [43]. The free amines of oligomeric DOCAs were conjugated with the thiol group of GLP-1A-Cys through a maleimide reaction. As a result, an oral chimeric GLP-1A was designed with GLP-1R agonist and oral delivery functions by maintaining the *n*-terminal region in exenatide for binding GLP-1R and targeting the *c*-terminal region for targeting ASBT.

ASBT in the intestine is used to transport bile acids by binding and facilitating transformation. In previous studies, oligomeric bile acids showed high binding affinity to ASBT, and macromolecule oral delivery using these oligomers demonstrated high oral bioavailability [43, 45, 56]. However, the pore size of ASBT made it difficult to transport high-MW oligomeric bile acids, and they stayed occluded in the bound state for endocytosis. This study focused on how oligomeric DOCA-conjugated GLP-1As bind and interact in silico, and which oligomeric DOCA-GLP-1As are favorable for transporter-mediated endocytosis. First, molecular docking analysis was used to confirm how oligomeric DOCAs bind to the binding cavity of ASBT (Fig. 1A and B). Although the binding energy decreased as the number of bile acids increased, *bis*DOCA showed the lowest estimated Ki value (Fig. S1). Thus, oligomeric DOCAs showed high binding affinity to ASBT, and their conjugation with GLP-1A demonstrates the potential for oral delivery of GLP-1A. MD simulation was conducted on the molecule that bound oligomeric DOCAs and oligomeric DOCA-G1A. During observation from baseline to 100 ps of the binding simulation between oligomeric DOCAs and ASBT protein, *bis*DOCA (− 137.45 kcal/mol) and *tetra*DOCA (− 148.27 kcal/mol) showed more stable interactions than *mono*DOCA (− 95.62 kcal/mol) (Fig. 1D). Furthermore, *tetra*DOCA became stable more quickly than *bis*DOCA (Fig. 1D). Stabilized binding poses described that *mono*DOCA and *bis*DOCA were observed inside the binding cavity, but two bile acids of *tetra*DOCA were located outside the binding cavity. The binding cavity of ASBT was large enough to accommodate *mono*DOCA and *bis*DOCA, but *tetra*DOCA was larger than the binding cavity (Fig. 1E). This showed that while *mono*DOCA and *bis*DOCA bound inside the binding cavity, *tetra*DOCA bound outside the binding cavity, thus affecting interaction stability. The total number of interaction bonds involved in binding was highest for *bis*DOCA and lowest for *mono*DOCA (Fig. S2). *mono*DOCA had more hydrogen bonds and fewer alkyl bonds than *bis*DOCA and *tetra*DOCA, which is related to the strong binding affinity of *bis*DOCA (Fig. 1F). However, the greater stability of *tetra*DOCA than *bis*DOCA is explained by the fact that *tetra*DOCA has more hydrogen bonds than *bis*DOCA, and more alkyl bonds. In other words, *tetra*DOCA exhibits stronger and more stable binding than other oligomeric DOCAs despite the limited binding cavity of ASBT.

MD simulations between oligomeric DOCA-G1A and ASBT protein were performed. The results indicated that GLP-1A peptide alone did not significantly interact with ASBT protein, but when conjugated with oligomeric DOCAs, it remained stably bound to the protein throughout the 100 ps simulation (Fig. 2A and B). The interaction energies of oligomeric DOCA-G1A were higher than those of GLP-1A alone, and the trend of interaction energies was the same as the molecular docking results (Figs. 1D and 2C), with *t*D-G1A showing the lowest energy and strongest interaction. Furthermore, the conformation of each DOCA motif was investigated to understand its interaction with the ASBT binding site. Both DOCA motifs in *b*D-G1A contributed to the binding through strong hydrophobic interactions. However, in *t*D-G1A with four DOCA motifs, only two motifs were actively involved in the binding through van der Waals interactions; the other DOCA motifs likely participated in the binding through electrostatic or weaker hydrophobic interactions with the ASBT protein (Fig. 2D). Overall, these results suggest that the presence of oligomeric DOCAs enhances the interaction between GLP-1A peptide and ASBT protein, and that the number and position of DOCA motifs play a significant role in the binding.

Chimeric GLP-1A was synthesized to confirm the possibility of establishing oral GLP-1A through MD simulation. We hypothesized that oral chimeric GLP-1A maintains the sequence related to GLP-1R binding and activation, while also having the ability to specifically target ASBT. Oligomeric DOCA-G1As (*m*D-G1A, *b*D-G1A, and *t*D-G1A) were synthesized through a maleimide reaction with the thiol group of GLP-1A-Cys (Fig. 3A). The oligomeric DOCA moiety allowed GLP-1A to bind with ASBT, and bile acids could thus be transported through ASBT without energy; however, oligomeric DOCA-G1As could not be transported because of their large molecular size. Instead, they created ASBT vesicles through the transporter-mediated endocytosis pathway, allowing them to be taken up by cells (Fig. 4A). The Caco-2 permeability results showed that the permeability to *t*D-G1A was improved compared with that to *m*D-G1A and *b*D-G1A (Fig. 4B). Furthermore, cellular transport of *t*D-G1A was inhibited using both Act D as ASBT inhibitor and/or CFZ as OST_α/β_ inhibitor, supporting that oligomeric DOCA-G1As facilitates ASBT-mediated transport (Fig. 4C). Similarly, the MD simulation results showed that high ASBT binding improved cellular permeability. The western blotting results showed that the ASBT band on the membrane moved to the cytoplasm when *t*D-G1A was applied, showing that *t*D-G1A utilized ASBT-mediated endocytosis (Fig. 4D and E). Based on the finding that GLP-1A-Cys showed the same GLP-1R binding affinity as exenatide, the binding affinity of oligomeric DOCA-G1As was evaluated (Fig. 4F). Insulin secretion by oligomeric DOCA-G1As was investigated to determine its binding to GLP-1R. Because GLP-1A secretes insulin in a glucose-dependent manner [57], insulin secretion was compared between low- and high-glucose conditions. The insulin secretion under high-glucose conditions was similar for both exenatide and GLP-1A-Cys, and oligomeric DOCA-G1As also exhibited an insulin secretion index comparable to that of exenatide (Fig. 4G and H). Therefore, oligomeric DOCA-G1As are unaffected by the activation of GLP-1R.

After designing chimeric GLP-1As in silico, the increase in cellular permeability after in vitro and in vivo oral absorption was compared. Orally administered GLP-1A-Cys showed low absorption and bioavailability (Fig. 5A and Table 1). Peptide stability within the stomach was critical for oral absorption because of the presence of gastric enzymes. Therefore, oral GLP-1As were administered after antacid treatment to confirm in vivo cellular absorption while excluding the stability issue in low gastric pH. T_max_ appeared within 30 min in the drug treatment group, indicating rapid absorption through transporters in the intestine. In the PK profile, *m*D-G1A showed a two-peak absorption pattern (Fig. 5B), which is commonly seen in formulations that use bile acid transporters (initial rapid absorption followed by reabsorption). Additionally, the flip-flop PK profile of *b*D-G1A explained the absorption within the first 30 min and reabsorption at 1.5 h (Fig. 5B). The high bioavailability of *t*D-G1A explained the impact of strong ASBT binding on absorption (Table 1). Intravenous GLP-1A-Cys rapidly decomposed within 6 h and needed to be administrated twice-a-day. However, all drug administration groups showed a two-compartment model with initial rapid absorption and slow elimination after 2 h, maintaining the drug concentration up to 6 h. Consequently, it is necessary to confirm whether the high T_max_ of *t*D-G1A or flip-flop PK profile of *b*D-G1A impacts the hypoglycemia effect. Despite *b*D-G1A and *t*D-G1A showing similar improvement of exposure (area under the curve and bioavailability), further investigation is needed.

GLP-1A exhibits a glucose-dependent hypoglycemic effect, i.e., shows a strong effect at higher blood glucose levels. In the diabetic disease model (*db/db* mice) of this study, diabetic mice had low tolerance to insulin and high plasma glucose levels even under fasting conditions. An IPGTT was conducted to measure the hypoglycemic effect of *b*D-G1A and *t*D-G1A, which had high oral absorption according to the PK results. Because oral GLP-1A can be affected by food, the drugs were administrated in a fasting state and glucose was administrated intraperitoneally to measure changes in the fasting glucose level. Unlike the similar exposure seen in the PK results, *t*D-G1A had a stronger hypoglycemic effect than *b*D-G1A. The rapid glucose-lowering effect within 1 h in *t*D-G1A was induced by fast absorption. *t*D-G1A lowered the glucose level to 89.7 mg/mL at 3 h while *b*D-G1A lowered it to 186.7 mg/mL, indicating that *t*D-G1A, which showed a higher C_max_ than *b*D-G1A even with the same exposure, had stronger effects (Fig. 6A). In other words, the blood glucose-lowering effect is influenced by both high exposure and high C_max_ following oral administration, with C_max_ having a greater influence than its profile.

To compare the effects of the drugs in a late diabetes-induced mouse model, we specifically selected *db*/*db* mice with a fasting glucose level > 500 mg/dL. The changes in glucose level induced by oral administration of *b*D-G1A or *t*D-G1A in diabetic mice were compared with changes induced by oral GLP-1A-Cys and subcutaneous exenatide. During the IPGTT, the hypoglycemic effect of *t*D-G1A was greater; at higher glucose levels, the effect was similar, suggesting that the effect of GLP-1A is more pronounced under high-glucose conditions. Despite the short half-life of chimeric GLP-1A in terms of pharmacokinetics, the hypoglycemic effects of *b*D-G1A and *t*D-G1A began within 1 h after drug administration and persisted for up to 6 h, as demonstrated by pharmacodynamic analysis (Fig. 6B). This finding can be attributed to the ability of GLP-1A to indirectly lower blood glucose levels through insulin; the duration of insulin action can reach 6 h. Consequently, *b*D-G1A and *t*D-G1A were able to maintain their effects for a considerable duration. However, both *b*D-G1A and *t*D-G1A could only lower glucose levels to 200 mg/dL in the late diabetic model, which led to reduced insulin tolerance. In contrast, subcutaneous exenatide was able to lower glucose levels to 100 mg/dL. Thus, the hypoglycemic effect of *t*D-G1A was reduced in the context of late-stage diabetes. This inherent limitation of oral oligomeric DOCA-G1As highlights the importance of focusing on prevention, rather than treatment, of severe diabetes through oral GLP-1A administration.

## Conclusion

This study proposes the design of chimeric GLP-1A that utilizes transporter-mediated endocytosis for oral delivery. Oral GLP-1A has been limited by its large MW and high hydrophilicity, making it difficult to penetrate hydrophobic membranes and resulting in low cellular permeability. Therefore, we designed a conjugation of oligomeric DOCAs with GLP-1A to utilize ASBT-mediated endocytosis, which enhances its oral bioavailability. The in silico molecular docking and MD simulation results suggested that *tetra*DOCA-G1A was the most favorable oligomeric DOCA for transporter-mediated endocytosis of chimeric GLP-1A. The in vitro*/*in vivo results proved that oligomeric DOCA improved cellular permeability and oral bioavailability. In conclusion, this oral chimeric GLP-1A strategy suggests that applying oligomeric DOCAs as a carrier for oral administration of GLP-1A is a viable approach to increase its oral bioavailability and improve the treatment of diabetes. This is a promising solution for enhancing the effectiveness of oral GLP-1A.

### Supplementary Information

**Additional file 1: Fig. S1.** *In silico* molecular docking analysis for estimated Ki and binding energy of oligomeric DOCAs to ASBT. **Fig. S2.** **A** Residue interacting of *mono*DOCA and *bis*DOCA to the ASBT binding cavity, and the type of interaction. **B** Overall interactions between oligomeric DOCAs and ASBT. **Fig. S3.** Ligand RMSD of oligomeric DOCA-G1A during 100 ps of MD simulation. **Fig. S4.** The DOCA motif region of *m*D-G1A that directly interacts with the ASBT binding site for motif-specific interaction energy calculation. **Fig. S5.** 1H-NMR (500 MHz) of the oligomeric DOCAs. **A** *mono*DOCA, **B** *bis*DOCA, and **C** *tetra*DOCA. **Fig. S6.** 1H-NMR (500 MHz) of the oligomeric DOCA-EMCS conjugates. **A** *mono*DOCA-EMCS, **B** *bis*DOCA-EMCS, and **C** *tetra*DOCA-EMCS. **Fig. S7.** MALDI-TOF MS results of the oligomeric DOCA-EMCS conjugates (*mono*DOCA-EMCS, *bis*DOCA-EMCS, and *tetra*DOCA-EMCS).

## Data Availability

All data used in the study are presented in the manuscript and/or Supplementary Materials. Additional data related to this study may be requested from the authors.

## Abbreviations

4-MMP: 4-Methylmorpholine
ASBT: Apical sodium bile acid transporter
DCC: Dicyclohexylcarbodiimide
DOCA: Deoxycholic acid
DMEM: Dulbecco’s modified Eagle’s medium
DMF: *N,N*-Dimethylformamide
EMCS: 6-Mleimidohexanoic NHS
GBSW: Generalized born with simple switching
GI: Gastrointestinal
GLP-1A: Glucagon-like peptide 1 agonist
HBSS: Hank’s balanced salt solution
IPGTT: Intraperitoneal glucose tolerance test
KRBB: Krebs–Ringer bicarbonate buffer
MD: Molecular dynamics
MDCK: Madin–Darby canine kidney cells
PBS: Phosphate-buffered saline
PK: Pharmacokinetic
