# Supplementary figures and images for: Design of chimeric GLP-1A using oligomeric bile acids to utilize transporter-mediated endocytosis for oral delivery
Source: Biomater Res. 2023 Sep 2;27:83. doi: 10.1186/s40824-023-00421-7 (PMC10474648; doi:10.1186/s40824-023-00421-7)

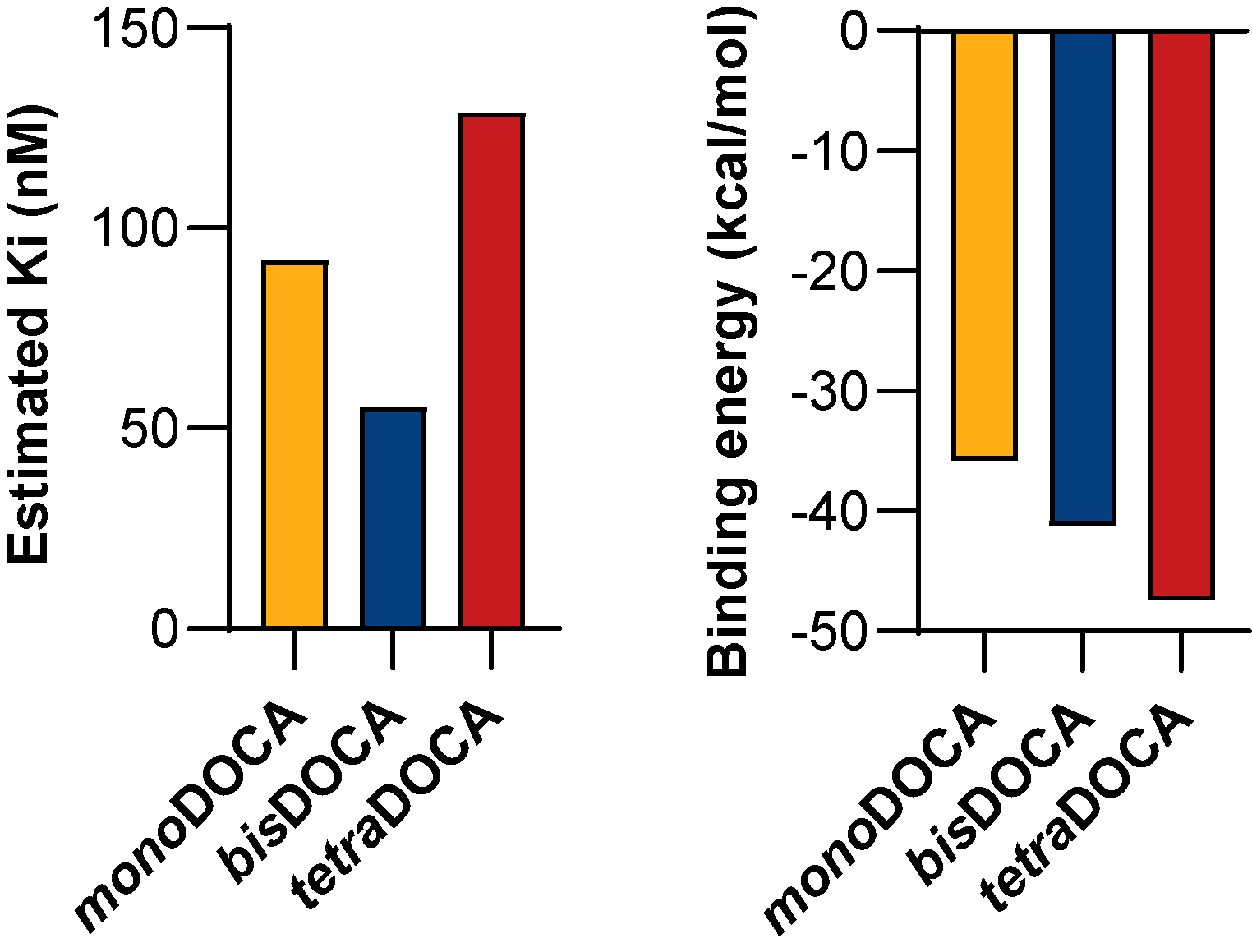

Supplement: Supplementary file 1 — Additional file 1: Fig. S1. In silico molecular docking analysis for estimated Ki and binding energy of oligomeric DOCAs to ASBT. Fig. S2. A Residue interacting of monoDOCA and bisDOCA to the ASBT binding cavity, and the type of interaction. B Overall interactions between oligomeric DOCAs and ASBT. Fig. S3. Ligand RMSD of oligomeric DOCA-G1A during 100 ps of MD simulation. Fig. S4. The DOCA motif region of mD-G1A that directly interacts with the ASBT binding site for motif-specific interaction energy calculation. Fig. S5. 1H-NMR (500 MHz) of the oligomeric DOCAs. A monoDOCA, B bisDOCA, and C tetraDOCA. Fig. S6. 1H-NMR (500 MHz) of the oligomeric DOCA-EMCS conjugates. A monoDOCA-EMCS, B bisDOCA-EMCS, and C tetraDOCA-EMCS. Fig. S7. MALDI-TOF MS results of the oligomeric DOCA-EMCS conjugates (monoDOCA-EMCS, bisDOCA-EMCS, and tetraDOCA-EMCS). [file 40824_2023_421_MOESM1_ESM.zip › Supplementary Figure 1.jpg]

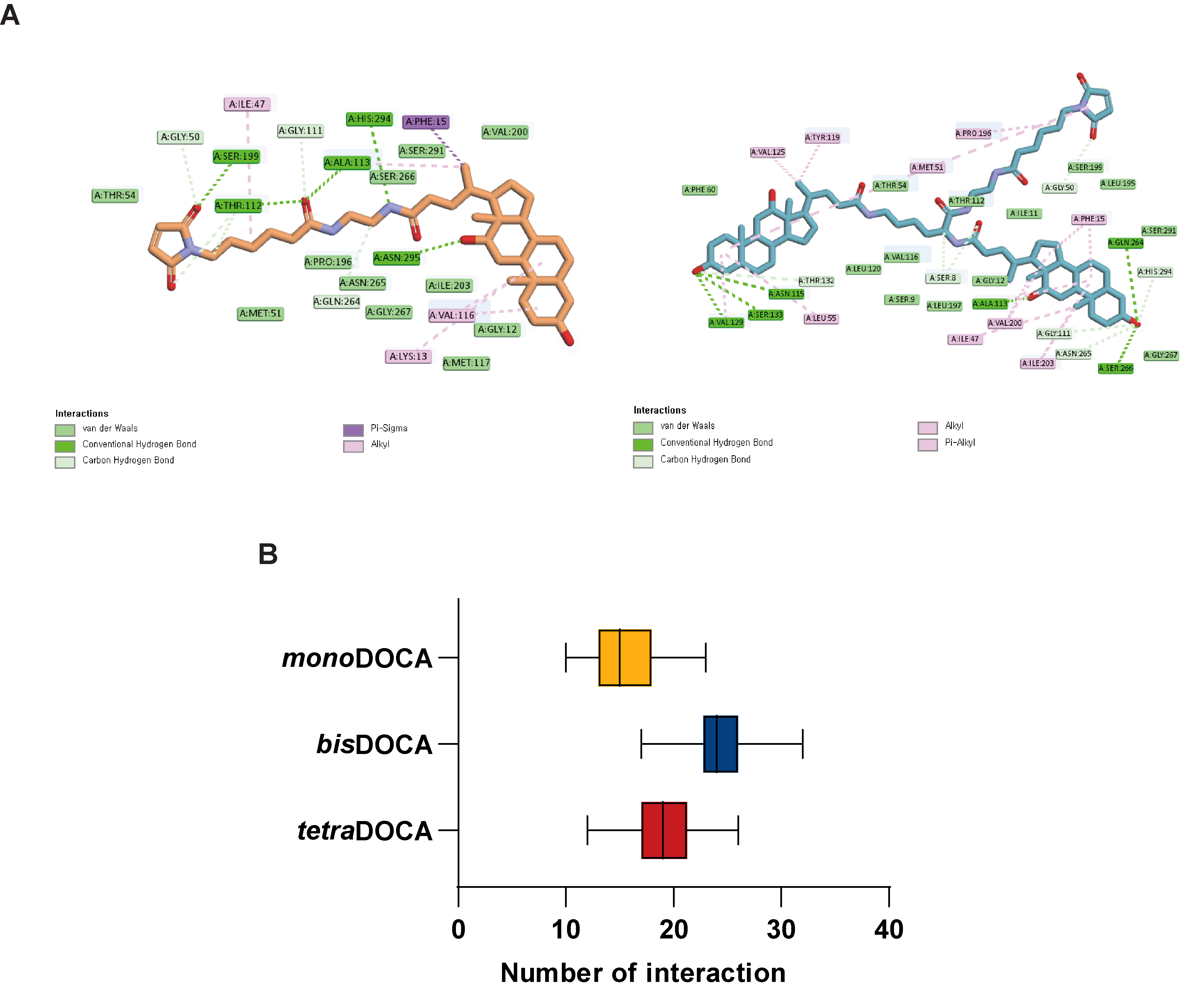

Supplement: Supplementary file 1 — Additional file 1: Fig. S1. In silico molecular docking analysis for estimated Ki and binding energy of oligomeric DOCAs to ASBT. Fig. S2. A Residue interacting of monoDOCA and bisDOCA to the ASBT binding cavity, and the type of interaction. B Overall interactions between oligomeric DOCAs and ASBT. Fig. S3. Ligand RMSD of oligomeric DOCA-G1A during 100 ps of MD simulation. Fig. S4. The DOCA motif region of mD-G1A that directly interacts with the ASBT binding site for motif-specific interaction energy calculation. Fig. S5. 1H-NMR (500 MHz) of the oligomeric DOCAs. A monoDOCA, B bisDOCA, and C tetraDOCA. Fig. S6. 1H-NMR (500 MHz) of the oligomeric DOCA-EMCS conjugates. A monoDOCA-EMCS, B bisDOCA-EMCS, and C tetraDOCA-EMCS. Fig. S7. MALDI-TOF MS results of the oligomeric DOCA-EMCS conjugates (monoDOCA-EMCS, bisDOCA-EMCS, and tetraDOCA-EMCS). [file 40824_2023_421_MOESM1_ESM.zip › Supplementary Figure 2.jpg]

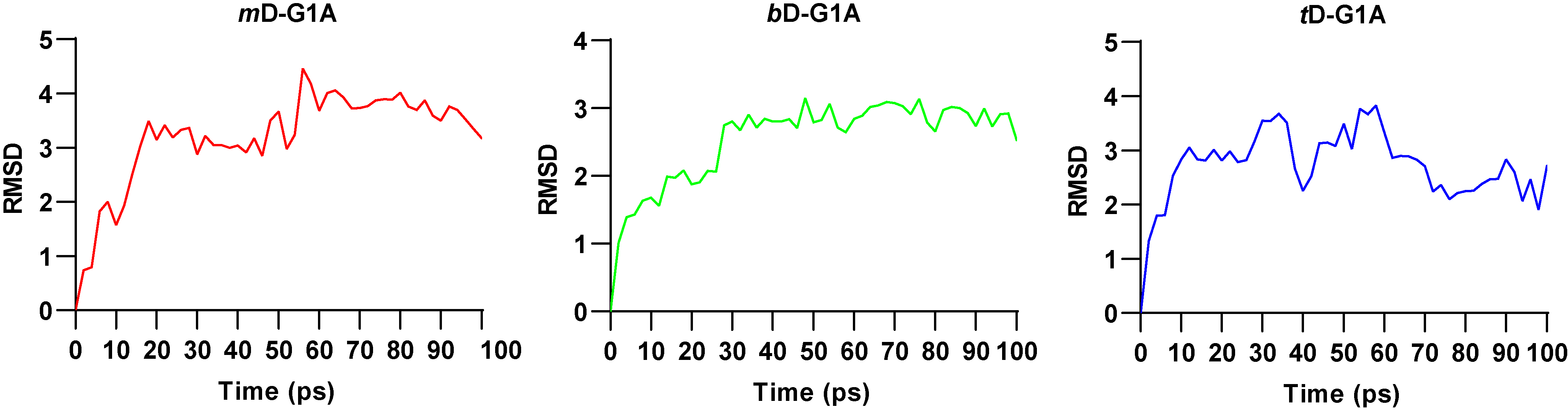

Supplement: Supplementary file 1 — Additional file 1: Fig. S1. In silico molecular docking analysis for estimated Ki and binding energy of oligomeric DOCAs to ASBT. Fig. S2. A Residue interacting of monoDOCA and bisDOCA to the ASBT binding cavity, and the type of interaction. B Overall interactions between oligomeric DOCAs and ASBT. Fig. S3. Ligand RMSD of oligomeric DOCA-G1A during 100 ps of MD simulation. Fig. S4. The DOCA motif region of mD-G1A that directly interacts with the ASBT binding site for motif-specific interaction energy calculation. Fig. S5. 1H-NMR (500 MHz) of the oligomeric DOCAs. A monoDOCA, B bisDOCA, and C tetraDOCA. Fig. S6. 1H-NMR (500 MHz) of the oligomeric DOCA-EMCS conjugates. A monoDOCA-EMCS, B bisDOCA-EMCS, and C tetraDOCA-EMCS. Fig. S7. MALDI-TOF MS results of the oligomeric DOCA-EMCS conjugates (monoDOCA-EMCS, bisDOCA-EMCS, and tetraDOCA-EMCS). [file 40824_2023_421_MOESM1_ESM.zip › Supplementary Figure 3.jpg]

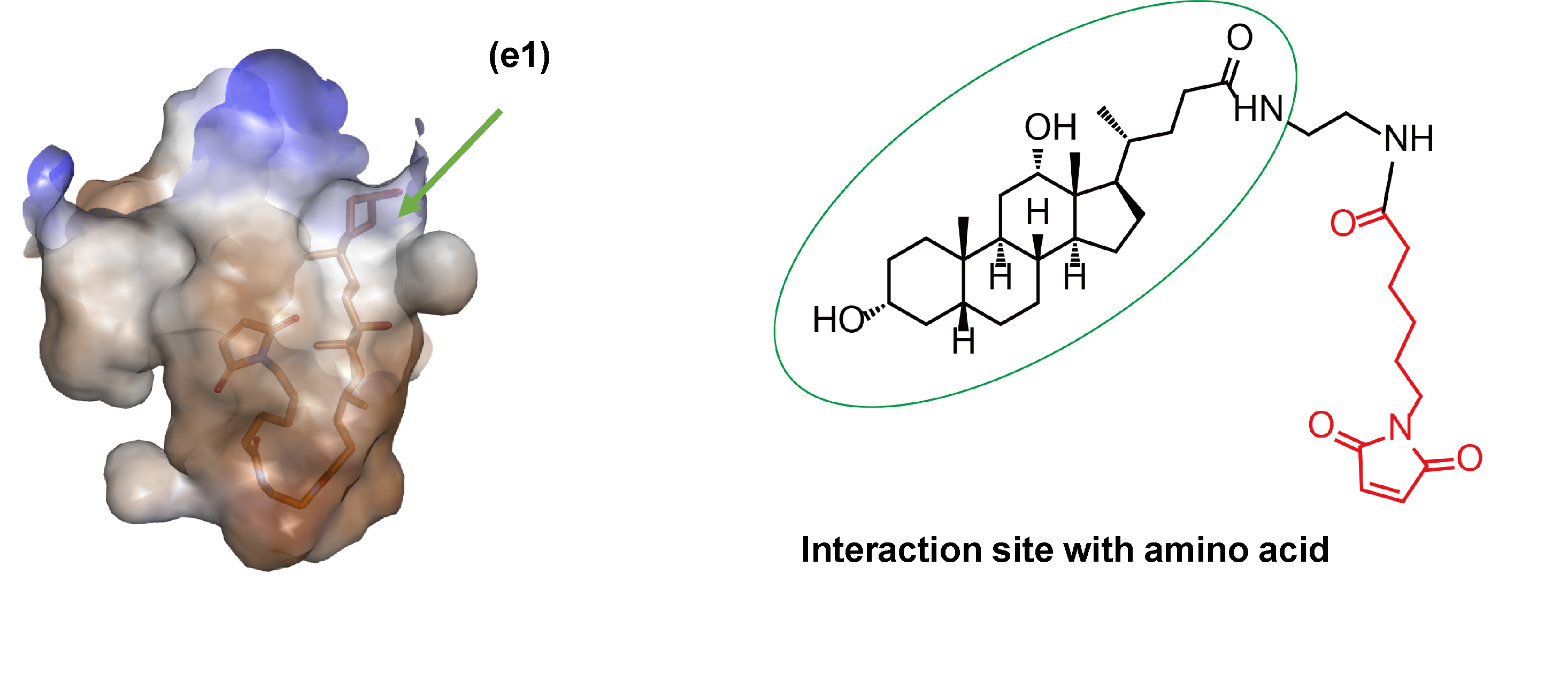

Supplement: Supplementary file 1 — Additional file 1: Fig. S1. In silico molecular docking analysis for estimated Ki and binding energy of oligomeric DOCAs to ASBT. Fig. S2. A Residue interacting of monoDOCA and bisDOCA to the ASBT binding cavity, and the type of interaction. B Overall interactions between oligomeric DOCAs and ASBT. Fig. S3. Ligand RMSD of oligomeric DOCA-G1A during 100 ps of MD simulation. Fig. S4. The DOCA motif region of mD-G1A that directly interacts with the ASBT binding site for motif-specific interaction energy calculation. Fig. S5. 1H-NMR (500 MHz) of the oligomeric DOCAs. A monoDOCA, B bisDOCA, and C tetraDOCA. Fig. S6. 1H-NMR (500 MHz) of the oligomeric DOCA-EMCS conjugates. A monoDOCA-EMCS, B bisDOCA-EMCS, and C tetraDOCA-EMCS. Fig. S7. MALDI-TOF MS results of the oligomeric DOCA-EMCS conjugates (monoDOCA-EMCS, bisDOCA-EMCS, and tetraDOCA-EMCS). [file 40824_2023_421_MOESM1_ESM.zip › Supplementary Figure 4.jpg]

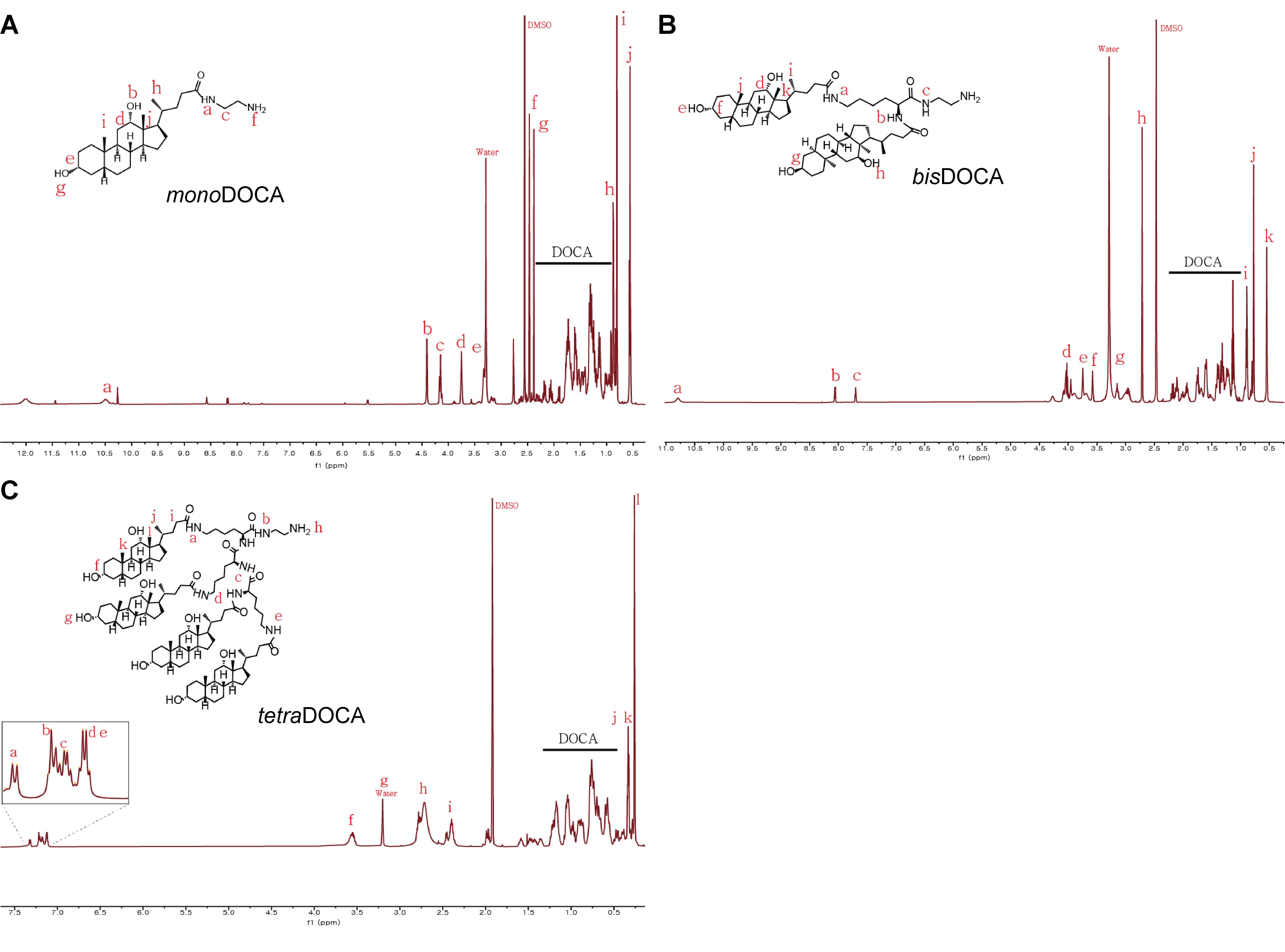

Supplement: Supplementary file 1 — Additional file 1: Fig. S1. In silico molecular docking analysis for estimated Ki and binding energy of oligomeric DOCAs to ASBT. Fig. S2. A Residue interacting of monoDOCA and bisDOCA to the ASBT binding cavity, and the type of interaction. B Overall interactions between oligomeric DOCAs and ASBT. Fig. S3. Ligand RMSD of oligomeric DOCA-G1A during 100 ps of MD simulation. Fig. S4. The DOCA motif region of mD-G1A that directly interacts with the ASBT binding site for motif-specific interaction energy calculation. Fig. S5. 1H-NMR (500 MHz) of the oligomeric DOCAs. A monoDOCA, B bisDOCA, and C tetraDOCA. Fig. S6. 1H-NMR (500 MHz) of the oligomeric DOCA-EMCS conjugates. A monoDOCA-EMCS, B bisDOCA-EMCS, and C tetraDOCA-EMCS. Fig. S7. MALDI-TOF MS results of the oligomeric DOCA-EMCS conjugates (monoDOCA-EMCS, bisDOCA-EMCS, and tetraDOCA-EMCS). [file 40824_2023_421_MOESM1_ESM.zip › Supplementary Figure 5.jpg]

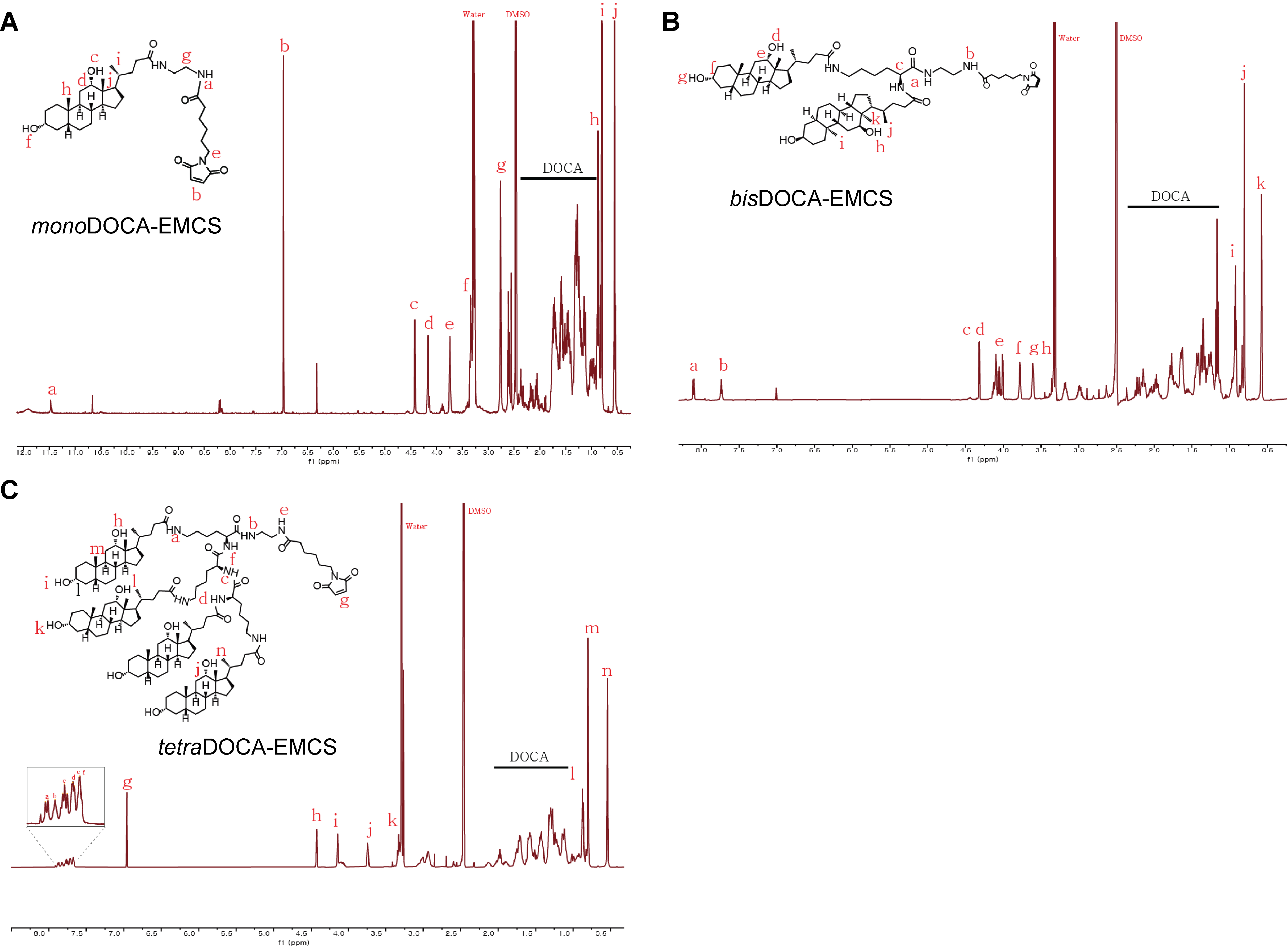

Supplement: Supplementary file 1 — Additional file 1: Fig. S1. In silico molecular docking analysis for estimated Ki and binding energy of oligomeric DOCAs to ASBT. Fig. S2. A Residue interacting of monoDOCA and bisDOCA to the ASBT binding cavity, and the type of interaction. B Overall interactions between oligomeric DOCAs and ASBT. Fig. S3. Ligand RMSD of oligomeric DOCA-G1A during 100 ps of MD simulation. Fig. S4. The DOCA motif region of mD-G1A that directly interacts with the ASBT binding site for motif-specific interaction energy calculation. Fig. S5. 1H-NMR (500 MHz) of the oligomeric DOCAs. A monoDOCA, B bisDOCA, and C tetraDOCA. Fig. S6. 1H-NMR (500 MHz) of the oligomeric DOCA-EMCS conjugates. A monoDOCA-EMCS, B bisDOCA-EMCS, and C tetraDOCA-EMCS. Fig. S7. MALDI-TOF MS results of the oligomeric DOCA-EMCS conjugates (monoDOCA-EMCS, bisDOCA-EMCS, and tetraDOCA-EMCS). [file 40824_2023_421_MOESM1_ESM.zip › Supplementary Figure 6.jpg]

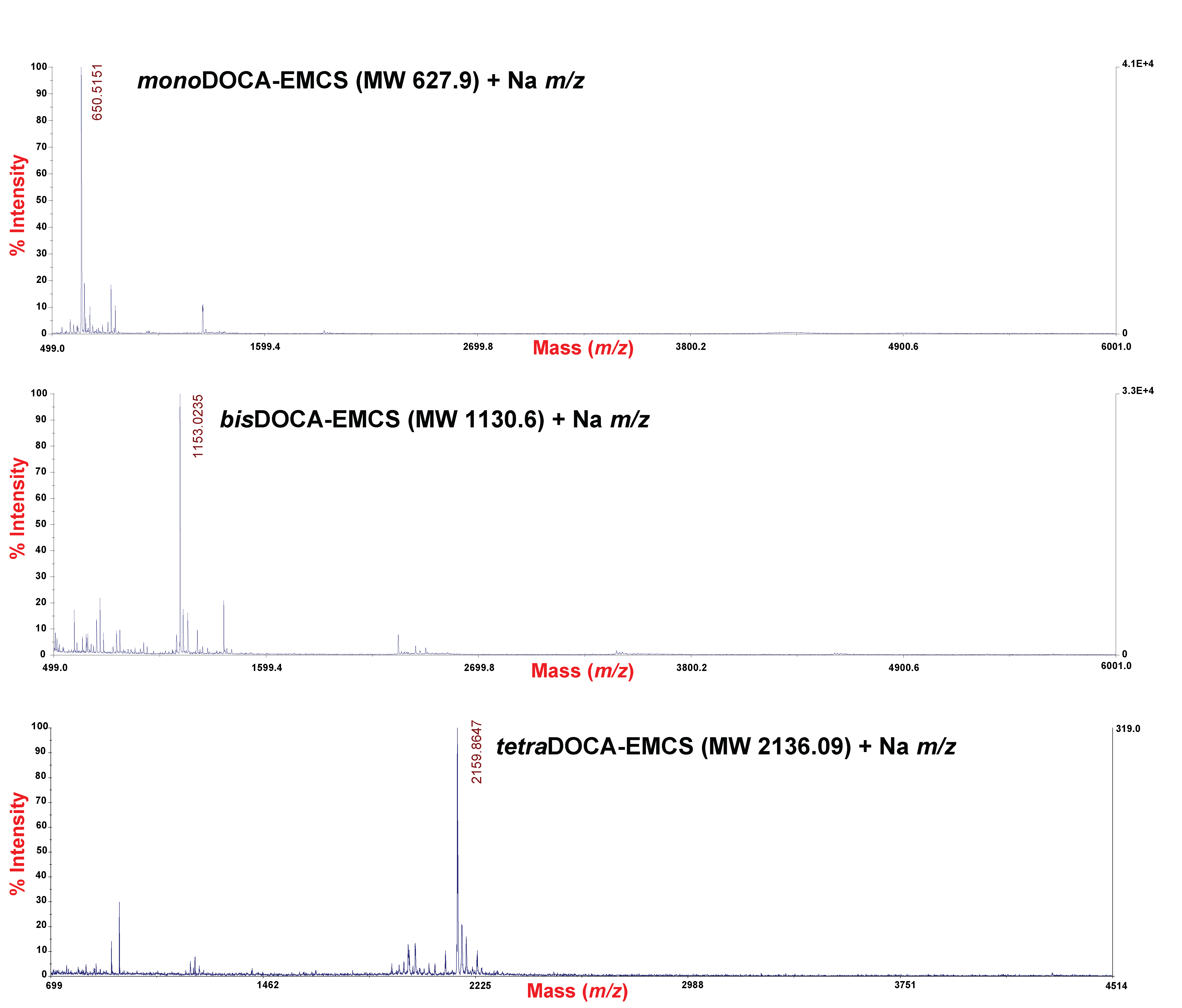

Supplement: Supplementary file 1 — Additional file 1: Fig. S1. In silico molecular docking analysis for estimated Ki and binding energy of oligomeric DOCAs to ASBT. Fig. S2. A Residue interacting of monoDOCA and bisDOCA to the ASBT binding cavity, and the type of interaction. B Overall interactions between oligomeric DOCAs and ASBT. Fig. S3. Ligand RMSD of oligomeric DOCA-G1A during 100 ps of MD simulation. Fig. S4. The DOCA motif region of mD-G1A that directly interacts with the ASBT binding site for motif-specific interaction energy calculation. Fig. S5. 1H-NMR (500 MHz) of the oligomeric DOCAs. A monoDOCA, B bisDOCA, and C tetraDOCA. Fig. S6. 1H-NMR (500 MHz) of the oligomeric DOCA-EMCS conjugates. A monoDOCA-EMCS, B bisDOCA-EMCS, and C tetraDOCA-EMCS. Fig. S7. MALDI-TOF MS results of the oligomeric DOCA-EMCS conjugates (monoDOCA-EMCS, bisDOCA-EMCS, and tetraDOCA-EMCS). [file 40824_2023_421_MOESM1_ESM.zip › Supplementary Figure 7.jpg]
